# Experiences and perceptions of sleep quality among older adults in Bangka Belitung, Indonesia: a qualitative study

**DOI:** 10.1590/1980-220X-REEUSP-2026-0008en

**Published:** 2026-09-07

**Authors:** Indah Permata Sari, Tita Hariyanti, Retno Lestrari, Respati Suryanto Dradjat

**Affiliations:** 1 Universitas Brawijaya, Faculty of Medicine, Doctoral Program in Medical Science, Malang, Indonesia.; 2 Institut Citra Internasional, Faculty of Nursing, Department of Nursing, Pangkalpinang, Indonesia.; 3 Universitas Brawijaya, Faculty of Medicine, Department of Public Health, Malang, Indonesia.; 4 Universitas Brawijaya, Faculty of Health Sciences, Department of Nursing, Malang, Indonesia.; 5 Universitas Brawijaya, Faculty of Medicine, Department of Orthopaedic and Traumatology, Malang, Indonesia.; 6 Dr. Saiful Anwar Regional General Hospital, Department of Orthopaedic and Traumatology, Malang, Indonesia.

**Keywords:** Sleep Wake Disorders, Aged, Sleep Quality, Qualitative Research, Coping Skills, Transtornos do Sono-Vigília, Idosos, Qualidade do Sono, Pesquisa Qualitativa, Habilidades de Enfrentamento

## Abstract

**Objective:**

To explore older adults’ perceptions and experiences of sleep and strategies used to cope with poor sleep quality.

**Method:**

A qualitative descriptive study was conducted with 27 purposively selected older adults from community and institutional settings in Bangka Belitung. Data were collected from June to August 2025 through in-depth interviews and analyzed thematically with triangulation, member checking, and adherence to COREQ guidelines.

**Results:**

Participants were mostly women aged 60–74 years, many widowed and with chronic conditions. Two themes emerged: poor sleep experiences and coping strategies. Common problems included difficulty initiating and maintaining sleep, nighttime awakenings, early awakening, and daytime fatigue. Sleep disruption was linked to psychological distress and environmental discomfort. Coping strategies included behavioral changes, spiritual practices, daytime naps, and activity adjustment.

**Conclusion:**

The findings suggest that sleep among participating older adults in Bangka Belitung is perceived as multidimensional, shaped by physiological, psychological, environmental, and spiritual factors, highlighting the need for culturally sensitive, community-based support beyond self-initiated strategies.

## RESUMO

**Objetivo:**

Explorar as percepções e experiências de idosos em relação ao sono e às estratégias utilizadas para lidar com sua má qualidade.

**Método:**

Foi realizado um estudo descritivo qualitativo com 27 idosos selecionados intencionalmente, provenientes de comunidades e instituições em Bangka Belitung. Os dados foram coletados de junho a agosto de 2025 por meio de entrevistas em profundidade e analisados tematicamente com triangulação, verificação com os participantes e adesão às diretrizes do COREQ.

**Resultados:**

Os participantes eram, em sua maioria, mulheres com idades entre 60 e 74 anos, muitas viúvas e com doenças crônicas. Dois temas emergiram: experiências ruins de sono e estratégias de enfrentamento. Os problemas mais comuns incluíam dificuldade em iniciar e manter o sono, despertares noturnos, despertar precoce e fadiga diurna. A perturbação do sono foi associada a angústia psicológica e desconforto ambiental. As estratégias de enfrentamento incluíram mudanças comportamentais, práticas espirituais, cochilos diurnos e ajustes nas atividades.

**Conclusão:**

Os resultados deste estudo sugerem que o sono entre os idosos participantes em Bangka Belitung é percebido como multidimensional, moldado por fatores fisiológicos, psicológicos, ambientais e espirituais, destacando-se a necessidade de apoio comunitário e culturalmente sensível, que vá além de estratégias iniciadas individualmente.

## INTRODUCTION

Sleep quality is a central component of health and wellbeing in older adults, influencing physical functioning, cognition, emotional regulation, and overall quality of life^(1,2)^. Aging is accompanied by physiological changes in sleep architecture, such as shorter sleep duration, increased nighttime awakenings, and reduced deep sleep^(3)^. However, sleep in later life is not shaped by biology alone; social roles, daily routines, living arrangements, and cultural norms also contribute to how older adults experience and interpret sleep^(4,5,6)^. In many communities, sleep problems in older adults are normalized as a natural part of aging and remain underreported. As a result, the subjective meaning of “good” or “poor” sleep among older adults often differs from clinical definitions.

Understanding sleep quality in later life can be approached not only through symptoms and clinical indicators, but also through how older adults conceptualize and make sense of their sleep^(7)^. While many previous studies have focused primarily on quantitative assessments and measurable outcomes such as Pittsburgh Sleep Quality Index (PSQI) scores, sleep duration, and sleep efficiency, fewer have examined the subjective meanings and interpretations attached to sleep in old age^(8)^. Qualitative approaches offer valuable insight into how expectations about aging, health conditions, emotional states, family interaction, and environmental contexts may intersect with sleep experiences^(9)^. Such perspectives are particularly relevant in settings where social and cultural contexts may influence beliefs about sleep and aging. Examining these dimensions may therefore contribute to a more contextualized theoretical understanding of sleep in later life^(10,11)^.

Bangka Belitung, an island province in Indonesia, represents a context in which sociocultural conditions may shape sleep-related experiences in older adulthood. Patterns of daily activity, communal living arrangements, religious practices, and island-based livelihoods may be related to sleep timing, rest opportunities, and nighttime awakenings. In addition, loneliness has emerged as an important issue affecting the well-being of older adults worldwide and may influence sleep experiences, particularly as cultural shifts and urbanization increase vulnerability to social isolation^(12)^. Although sleep problems among older adults have been widely studied, most previous research has primarily focused on quantitative indicators such as sleep duration, sleep efficiency, and PSQI scores. Previous evidence has also shown that sleep quality in older adults is influenced by multiple social and environmental factors, including family support, social relationships, and living conditions^(13)^. However, research examining how older adults subjectively perceive and interpret their sleep experiences within their sociocultural context remains limited. Studies have highlighted the importance of cultural perspectives in understanding sleep among older adults, yet qualitative evidence exploring the roles of family relationships, spirituality, environmental conditions, and cultural beliefs in shaping sleep experiences is still scarce, particularly in the Indonesian context. Furthermore, little is known about how older adults manage poor sleep using self-initiated coping strategies embedded within local sociocultural practices. This gap limits a comprehensive understanding of how sleep is integrated into everyday life and constrains the development of culturally sensitive interventions^(13,14)^. Therefore, this study aims to explore older adults’ perceptions and experiences of sleep and the strategies used to cope with poor sleep quality among older adults in Bangka Belitung. It is expected that these sleep experiences and coping strategies are closely linked with sociocultural norms, family relationships, environmental conditions, and spiritual practices.

## METHOD

### Design of Study

This study employed a qualitative descriptive design to explore sleep patterns and sleep quality among older adults. This approach enables comprehensive description of a phenomenon as narrated directly by participants with minimal prior theoretical interpretation. Qualitative inquiry emphasizes understanding human experiences and socially situated phenomena through rich, non-numerical accounts; therefore, it was considered appropriate for the present study^(15)^.

### Populasion and Local

The target population comprised older adults residing in Bangka Belitung Province, Indonesia. The province has approximately 50 community-based elderly health service centers and 2 residential elderly care facilities. Participants were recruited from selected sites that were chosen based on accessibility, participant availability, and the willingness of local staff to support the study. within the province between June and August 2025. The study focused on how sleep was experienced, interpreted, and managed by both community-dwelling and institutionalized older adults in these settings.

### Inclusion and Exclusion Criteria

Participants were included in the study if they were 60 years of age or older, reported experiencing sleep problems such as difficulty falling asleep, frequent nighttime awakenings, early morning awakening, short sleep duration, non-restorative sleep, or daytime dysfunction related to poor sleep, resided in the selected study settings, were able to communicate effectively during the interview, and provided written informed consent. Participants were excluded if they had severe medical illness, major physical limitations, or severe psychological disorders that could interfere with participation, or if they did not reside in the designated study locations. A total of 27 older adults participated in the study, including 22 participants living in the community through elderly integrated health service posts and 5 participants residing in elderly care facilities. Most community-dwelling participants lived with family members, while participants in residential care facilities shared living spaces with other residents without family relationships. These different living arrangements were considered important in understanding variations in sleep experiences, emotional support, and environmental comfort.

### Sample and Recruitment

Purposive sampling was used to recruit older adults who were considered capable of providing rich and detailed information regarding sleep experiences. To capture diverse perspectives, participants were recruited from different districts in Bangka Belitung and from both community and residential care settings. Recruitment was conducted through coordination with staff at the Elderly Integrated Health Posts and residential care facilities, who assisted in identifying eligible older adults based on the inclusion criteria. Final participant selection was conducted by the researcher. Interviews were continued until data saturation was reached, that is, when no new themes or relevant information emerged from subsequent interviews. A total of 27 older adults participated in the study. All participants who provided informed consent completed the interview, and no withdrawals occurred during data collection.

### Data Collection Procedure

Data were collected through individual, face-to-face in-depth interviews containing 32 open-ended questions that explored sleep experiences among older adults. The interviews were conducted by the principal researcher, who has a nursing background and experience in qualitative interviewing with older adults. The interview guide was developed using the PSQI framework originally introduced by Buysse et al.^(16)^ as a conceptual basis rather than as a standardized quantitative instrument. The guide was organized around seven dimensions derived from the PSQI and further expanded to reflect culturally relevant aspects of sleep among older adults in Indonesia, including psychological and emotional aspects, sleep patterns, behaviors and lifestyle, health status and physical conditions, external disturbances, characteristics of the physical sleep environment, and daytime dysfunction. Additional probing questions were included to explore family relationships, sociocultural norms, and spiritual practices related to sleep.

To ensure content validity and contextual relevance, the interview guide underwent expert judgment involving nursing experts, older adult care practitioners, and a psychology expert. Revisions were made based on their feedback regarding clarity, relevance, and cultural appropriateness of the questions. Interviews were conducted in participants’ homes for community-dwelling older adults and in private rooms within residential care facilities for institutionalized participants to ensure confidentiality and comfort. Each interview lasted approximately 45–60 minutes, was audio-recorded with the participant’s permission, and was supplemented by field notes documenting nonverbal expressions and contextual observations. No individuals other than the participant and the interviewer were present during the interviews. The complete interview guide is presented in Table 1.

**Table 1:** Interview guide for exploring sleep quality among older adults – Bangka Belitung, Indonesia, 2024.

No.	Interview question
**Section 1. Background information**	
1	Could you tell me a little about your daily life and routine activities?
2	Do you live alone, with a spouse, with children, or with other family members?
3	Do you have any chronic health conditions or take any regular medication?
**Section 2. Sleep habits and patterns**	
4	At what time do you usually go to bed at night? Is your bedtime regular?
5	At what time do you usually wake up in the morning? Do you wake up spontaneously or with help from others or an alarm?
6	Do you usually take naps during the day? If yes, how long do you usually sleep?
7	What is your usual bedtime routine? What do you normally do before going to sleep?
8	Have your sleep habits changed compared with a few years ago? What has changed and why?
**Section 3. Sleep environment**	
9	Do you sleep in a private room, with a spouse, or with other family members? Does this affect your sleep?
10	Do you find your mattress, pillow, and blanket comfortable? Do you feel pain or stiffness after sleeping?
11	How is the lighting in your bedroom when you sleep: dark, dim, or bright?
12	Do noises from outside or inside the house disturb your sleep?
13	How is the room temperature at night: hot, cold, or comfortable? Do you use a fan or air-conditioner?
14	How is the air quality in your room: humid, stuffy, or fresh? Do you open the window or use any device?
15	In your opinion, is there anything in your bedroom that could be improved to help you sleep better?
**Section 4. Lifestyle and psychological factors**	
16	Do you engage in physical activity or exercise? What kind and when?
17	Do you consume caffeine (coffee, tea, energy drinks) in the afternoon or at night?
18	Do you smoke or drink alcohol? If yes, is it close to bedtime?
19	Do you feel anxious, sad, or have many thoughts before sleeping? How does this affect your sleep?
20	Have you ever felt lonely or uneasy at night? How do you cope with it?
21	Do you use sleeping pills, supplements, or traditional remedies to help you sleep? If yes, how effective are they?
**Section 5. Perceptions and impact of sleep**	
22	Do you feel that your sleep is sufficient and of good quality?
23	Do you often feel tired, sleepy, or have difficulty concentrating during the day?
24	What does “good sleep” mean to you? What do you expect from a good night’s sleep?
25	What do you usually do when you cannot fall asleep? What helps you most?
26	Have you ever consulted a health professional about sleep problems? What advice were you given?
27	Do you think good sleep is important for your health? Why?
**Section 6. Spiritual, social, and ageing aspects**	
28	Do you have any spiritual practices before sleep (prayer, dhikr, meditation)? Do they help you sleep?
29	Has your sleep changed since becoming older? What are the main differences?
30	Does living with children or grandchildren affect your sleep pattern? In what ways?
**Closing questions**	
31	Is there anything else you would like to share about your sleep experience that we have not discussed?
32	In your opinion, what is the most important thing that helps older people sleep well at night?

### Data Analysis

Data were analyzed using thematic analysis following the framework proposed by Braun and Clarke^(17)^. Audio-recorded interviews were transcribed verbatim and read repeatedly to achieve familiarity with the data. Transcripts and field notes documenting nonverbal expressions were imported into NVivo 12 software to facilitate data management. Meaningful segments of text were identified, condensed into meaning units, coded, and subsequently grouped into categories and overarching themes reflecting recurring patterns in participants’ narratives. The analysis was iterative, involving constant comparison between raw data, codes, categories, and themes to maintain closeness to participants’ accounts. Triangulation was achieved using interview transcripts, field notes, and reflexive notes, while member checking was conducted with 5 participants from both community and institutional settings to confirm the credibility and consistency of the interpretations. Reflexive notes were maintained throughout to enhance awareness of researchers’ assumptions and minimize bias.

### Ethical Aspect

Ethical approval for this study was obtained from the Ethics Committee of Universitas Brawijaya (Approval No. 199/EC/KEPK-S3/07/2025). Written informed consent was obtained from all participants following explanation of the study’s aims, procedures, potential risks, and benefits. Confidentiality and anonymity were assured through the use of identification codes and secure storage of research materials. Participation was voluntary, and participants were informed of their right to withdraw at any time without negative consequences.

## RESULTS

### Participant Characteristics

The demographic characteristics of the participants are presented in Table 2. A total of 27 older adults participated in the study, including 22 from community settings and 5 from residential care facilities. Community-dwelling participants were recruited from four regencies, namely Bangka (n = 6), West Bangka (n = 5), Central Bangka (n = 5), and South Bangka (n = 4), as well as one municipality, Pangkalpinang (n = 2), while 5 participants were recruited from two residential elderly care facilities. Most of the participants were in the younger-old age group and the majority were women. Educational attainment was generally low to moderate, with most participants having completed primary or secondary education. More than half of the participants were widowed, and most were not formally employed, with many identifying themselves as housewives or retirees. A considerable proportion of participants reported having one or more chronic conditions. The most frequently reported health problems included hypertension and hypercholesterolemia, followed by diabetes and respiratory problems. Only a small number of participants reported no chronic illness.

**Table 2:** Demographic characteristics of participants in the qualitative study – Bangka Belitung, Indonesia, 2025 (n = 27).

Variable	Category	n (%)
Age	60–64 years	11 (40.7)
	65–69 years	7 (25.9)
	70–74 years	4 (14.8)
	≥ 75 years	5 (18.5)
Sex	Male	3 (11.1)
	Female	24 (88.9)
Highest Education Level	Primary school	10 (37.0)
	Junior high school	6 (22.2)
	Senior high school	9 (33.3)
	Diploma	2 (7.4)
Marital Status	Married	12 (44.4)
	Widowed	15 (55.6)
Current Occupation	Unemployed/housewife	15 (55.6)
	Casual laborer	1 (3.7)
	Trader/self-employed	5 (18.5)
	Farmer	1 (3.7)
	Retired	5 (18.5)
Medical History*	No chronic disease	9 (23.7)
	Hypertension	10 (26.3)
	Diabetes mellitus	4 (10.5)
	Hypercholesterolemia	8 (21.1)
	Gout	2 (5.3)
	Gastritis	2 (5.3)
	Respiratory disease/asthma	3 (7.9)

*Participants may report more than one chronic disease; therefore, the total number of responses for medical history was 38, and percentages were calculated based on n = 38.

The qualitative analysis generated two main themes with several related subthemes. The first theme reflected experiences of poor sleep quality, and the second described strategies used to manage sleep problems. These themes were derived from the coding and categorization process during data analysis. The themes and subthemes are outlined below, with illustrative participant quotations shown in Tables 3 and 4.

**Table 3:** Theme 1: experiences of poor sleep quality among older adults – Bangka Belitung, Indonesia, 2024.

Quotation		Subtheme		Category
“I usually go to sleep at 10 or half past 11.”		Habitual bedtime		Sleep schedule
“Sometimes I’m already asleep before 10.”		Variable bedtime		Sleep schedule
“I wake up around 3 a.m., spontaneously.”		Early morning awakening		Sleep fragmentation
“I wake up at 2–3 a.m. and then cannot fall back asleep.”		Difficulty returning to sleep		Sleep maintenance insomnia
“Since my fifties it has become difficult to fall asleep early.”		Change in sleep pattern with ageing		Sleep pattern change
“Sometimes I wake up to pray tahajud.”		Religious awakening at night		Nocturnal awakening
“I often wake up because the grandchild is crying.”		Awakened by others		Social/environmental disturbance
“If there is even a little noise, I wake up.”		Noise sensitivity		Sensory factor
“If it is hot, I cannot sleep.”		Heat intolerance		Environmental factor
“When the weather is cold, I often wake up.”		Cold sensitivity		Environmental factor
“Sometimes twice a week I find it hard to sleep.”		Episodic insomnia		Sleep disturbance
“I often feel sleepy during the day.”		Daytime sleepiness		Daytime consequence
“My body feels shaky when I lack sleep.”		Physical instability		Daytime consequence
“When I wake up, my back hurts.”		Morning pain		Physical symptom
“Sometimes I cannot concentrate.”		Reduced concentration		Cognitive consequence
“During the day I become lazy to work.”		Reduced productivity		Functional consequence
“My mood becomes easily angry.”		Irritability		Emotional consequence
“I worry about my grandchild being sick.”		Worry about family		Psychological factor
“I feel sad being alone at night.”		Loneliness		Emotional factor
“A lot on my mind… so I cannot sleep.”		Overthinking/rumination		Psychological factor
“I am afraid of the dark.”		Fear of darkness		Psychological factor
“I am afraid to be alone at home.”		Fear of being alone		Perceived insecurity
“I miss my spouse who has passed away.”		Grief		Emotional factor
“I cannot sleep if it’s dark.”		Light intolerance		Sleep environment
“The light must be dim.”		Light preference		Sleep environment
“It’s noisy, but I’m already used to it.”		Noise exposure		Sleep environment
“The mattress is comfortable.”		Mattress perception		Sleep environment
“The room is small but comfortable.”		Room comfort		Sleep environment
“Sleep gives you energy.”		Meaning of sleep		Perceived importance
“Sleep is important for health.”		Sleep and health		Perceived importance

**Table 4:** Theme 2: strategies used to manage sleep problems among older adults – Bangka Belitung, Indonesia, 2025.

Quotation		Subtheme		Category
“I pray before going to sleep.”		Bedtime ritual		Spiritual coping
“Reciting Yasin makes me sleepy on my own.”		Religious relaxation		Spiritual coping
“If I cannot sleep, I read the Qur’an.”		Religious strategy		Spiritual coping
“I drink milk.”		Pre-sleep consumption		Behavioral strategy
“Sometimes I watch TV.”		TV before sleeping		Media behavior
“I check WhatsApp group on my phone.”		Mobile phone use		Media behavior
“I lie down and relax first.”		Relaxation before sleep		Behavioral preparation
“I tidy up the house first.”		Light household activity		Behavioral coping
“I don’t have any special routine.”		Lack of sleep routine		Sleep behavior
“I take my blood pressure medicine.”		Medication use		Therapeutic strategy
“I like to just sit calmly first.”		Quiet sitting		Relaxation
“If I’m tired, only then I can nap.”		Compensatory nap		Restorative strategy
“Daytime napping is a must… otherwise I become cranky.”		Planned nap		Restorative strategy
“I try to control my thoughts.”		Thought control		Cognitive coping
“Don’t bring too many thoughts to bed.”		Cognitive reframing		Coping strategy
“Dhikr makes me calm.”		Emotional soothing		Spiritual coping

### Main Finding of the Study

The qualitative analysis generated two main themes with several related subthemes. The first theme reflected experiences of poor sleep quality, and the second described strategies used to manage sleep problems. These themes were derived from the coding and categorization process during data analysis. The themes and subthemes are outlined below, with illustrative participant quotations shown in Tables 3 and 4.

### Experiences of Poor Sleep Quality in Older Adults

#### Sleep Pattern in Older Adults

In general, the sleep patterns of older adults were characterized by relatively late bedtimes and short nighttime sleep duration. Many older adults reported going to bed between 22:00 and 23:00, and some were only able to fall asleep after midnight. One participant from Belinyu described that “sometimes it is after eleven when I can finally sleep… sometimes even in the middle of the night.” Another participant explained that this tendency to go to bed late had only emerged in recent years, stating that “since my fifties it has become difficult to fall asleep early.” Most participants also reported early morning awakenings, typically occurring between 02:00 and 05:00, often without any specific trigger. Several older adults stated that they had long become accustomed to waking up very early. As one participant expressed, “by four o’clock I am already awake and can no longer fall back asleep.” Among those residing in residential homes, very early awakening had become a part of their daily routine; one participant stated, “at two o’clock I am already awake… just sitting until morning.” When they reflected on earlier stages of life, participants perceived clear changes in their sleep. Many stated that they had slept much more soundly when they were younger. One elderly woman noted, “when I was young my sleep was good… now I often wake up in the middle of the night.” These changes were generally interpreted by participants as a natural part of the ageing process.

### Sleep Fragmentation and Nighttime Awakenings

Most older adults experienced fragmented sleep, characterized by frequent awakenings during the night and an inability to return to sleep afterward. Several older adults reported waking due to urination, bodily pain, or intrusive thoughts. One participant explained, “sometimes I wake up at one, then again at two… it is hard to fall back asleep until dawn.” Although many older adults perceived this condition as disturbing, they also described having gradually learned to accept it and adjust to it. Among those living in social care facilities, fragmented sleep was also commonly reported. One participant stated that after waking at two o’clock in the morning, they were unable to return to sleep: “I don’t know… I just stay in bed until morning.” Some participants woke up to perform night prayers (tahajud), but they did not always succeed in falling asleep again afterward. As one participant described, “sometimes I wake up at half past three for tahajud… but then it is difficult to sleep again.” The difficulty in returning to sleep often resulted in tiredness and fatigue during the day. Nevertheless, participants reported continuing to engage in their daily activities, such as working, preparing food, or selling goods.

### Psychological Contributors to Sleep Disturbance

Psychological factors emerged as one of the most prominent contributors to sleep disturbance.. Many participants reported that worries and intrusive thoughts greatly influenced their ability to sleep. One participant living in a social care facility, speaking in a sad tone, stated, “sometimes I feel sad… I think too much… I miss my children.” Another participant expressed deep concern about a grandchild with heart disease, saying, “I keep thinking about my grandchild… I’m afraid the treatment in Jakarta will not succeed.”

Several older adults experienced substantial family-related stress. One grandmother from Belinyu shared her experience of being refused by her daughter-in-law when she wished to live with her son, saying, “it’s not that I am not sad… but my son is afraid of his wife.” Such experiences intensified the emotional burden experienced by older adults and had a direct impact on their sleep quality. The influence of psychological distress was clearly reflected in the way participants described their sleep process. Some participants explained that when there was “too much on my mind, I simply cannot sleep,” while others reported lying down for a long time yet still being unable to close their eyes. These mental and emotional conditions served as major triggers of sleep difficulties among many participants. Various psychological experiences affecting sleep, such as anxiety, excessive rumination, and loneliness, as summarized in Table 3, illustrated the relationship between psychological factors and sleep quality among older adults.

### Environmental Contributors to Poor Sleep

Bedroom environmental conditions influenced sleep differently across participants. Preferences regarding lighting varied considerably. Some participants stated that they could only sleep when the light was fully on, whereas others reported that they were unable to sleep unless the room was completely dark. One older adult said, “it is usually dark… if it is bright I cannot sleep.” In contrast, another participant stated, “there must be a light… if it is dark I feel short of breath.” Room temperature was also described as an important factor. Many participants preferred electric fans and avoided air conditioners because they felt physically uncomfortable when exposed to cold air. One participant explained, “when using the air conditioner my bones hurt… but using a fan is fine.” Environmental noise was generally not perceived as a major disturbance by most participants. Several of them even reported that once they had fallen asleep, outside sounds were no longer noticed, as one participant remarked, “once I am asleep… I sleep… I do not hear anything anymore.” Most participants considered their mattresses and pillows comfortable. However, some older adults reported experiencing pain or stiffness upon waking, particularly among those who engaged in physically demanding work or who had joint problems.

### Daytime Consequences of Poor Sleep

Poor nighttime sleep resulted in a range of daytime consequences among participants. Several participants reported experiencing excessive daytime sleepiness, fatigue, and decreased concentration. One participant who worked in a social care facility stated, “during the day I often feel sleepy… tired… but I cannot take a nap because I have to work.” This situation suggests that limited opportunity for daytime rest may worsen the effects of inadequate nighttime sleep. In contrast, some older adults described that their bodies had already adapted to shorter nighttime sleep duration. One participant explained, “thank God it is enough… I usually wake up at four, and my body does not feel dizzy.” This suggests substantial individual variation in tolerance to sleep duration. A number of participants also relied on daytime napping as a compensatory strategy. Short naps lasting approximately 30 to 60 minutes were considered helpful for restoring energy and reducing fatigue throughout the day. This practice was especially common among participants who were unable to return to sleep after awakening during the night.

### Perceived Meaning and Importance of Sleep

Most participants perceived sleep as an essential element for both physical and emotional health. Sleep was perceived as a means of restoring energy after daily activities, as reflected in the statement of one older adult who said, “sleep replaces energy… so that the body feels fresh.” This understanding was evident in the way they evaluated their sleep quality, which was based more on how their bodies felt upon waking rather than solely on the duration of sleep.

Spiritual practices before sleep also played an important role in participants’ experiences of sleep. Prayers and dhikr, an Islamic spiritual practice involving remembrance of God through repetitive prayer or recitation, were considered helpful in calming the mind, reducing anxiety, and facilitating sleep onset. One participant explained, “I pray to be able to sleep soundly… so that when I wake up my body feels good.” Such spiritual activities formed part of their nightly routines and were perceived as having a soothing effect. Perceptions of ideal sleep duration varied among participants. Some older adults felt that short sleep was still sufficient and did not interfere with their daily activities, whereas others expressed a desire for longer sleep in order to feel more refreshed. This variation indicated that sleep experiences in older adults did not always align with general sleep standards, but were instead influenced by personal habits, health conditions, and individual tolerance to rest needs. The ways in which older adults perceived the functions of sleep, ideal sleep duration, and its relationship with health, as presented in Table 3, illustrated how participants evaluated the role of sleep in their daily lives.

### Strategies used to Manage Sleep Problems in Older Adults

#### Behavioral Strategies and Bedtime Routines

Most participants reported having specific habits that helped them prepare their bodies for sleep. Spiritual activities were the most commonly mentioned practices, including praying, reciting dhikr, and reading “Yasin” and “Al-Mulk”, which are chapters of the Holy Qur’an commonly recited by Muslims for spiritual comfort and relaxation. These activities were perceived as ways to calm the mind before sleep. One participant explained, “when it is hard to sleep, I read and recite dhikr… eventually I fall asleep on my own.” In addition to spiritual practices, some older adults engaged in simple routines such as sitting and relaxing, tidying the room, or drinking a glass of milk. Others Others reported watching television or using their mobile phones before bedtime, particularly those living with family members. In some cases, however, gadget use delayed bedtime, as expressed by one participant who said, “nowadays there are lots of WhatsApp groups… so before sleeping I use my phone first.” These various activities illustrated the diversity of ways in which older adults prepared themselves for sleep, with choices depending on personal habits, comfort, and daily living conditions.

### Cognitive and Emotional Coping Strategies

Cognitive and emotional coping strategies were frequently described by older adults as ways of managing sleep difficulties. Participants reported attempting to calm themselves, control excessive thoughts, and reduce rumination before sleep. Some consciously reminded themselves “not to think too much,” while others tried to redirect their attention when worries arose at night. Acceptance also emerged as an important coping strategy, whereby sleep problems were viewed as a natural consequence of ageing. This stance helped reduce frustration related to frequent awakenings or short sleep duration. Emotional coping was closely intertwined with spiritual meaning. Prayer and dhikr were perceived not only as habitual behaviors but also as ways to regulate emotions, surrender worries, and achieve peace of mind. Through this meaning-making process, sleep was understood as both a biological necessity and a source of renewed energy and emotional relief. These cognitive and emotional strategies illustrate how older adults actively adapted to poor sleep quality while maintaining psychological well-being.

### Physiological and Restorative Strategies

Physiological and restorative strategies were also reported by older adults as ways to compensate for poor nighttime sleep. Several participants described relying on short daytime naps to restore energy, especially when they were unable to return to sleep after awakening at night. Naps lasting about 30 to 60 minutes were perceived as sufficient to reduce fatigue, relieve bodily discomfort, and improve alertness during the day. Others reported adjusting daily activities, such as reducing physically demanding work or taking brief periods of rest, to prevent excessive tiredness. Some participants perceived that their bodies had gradually adapted to shorter sleep duration at night, suggesting physiological adaptation to altered sleep patterns. They reported waking early yet still feeling “fine” or not dizzy, indicating individual variability in sleep need and tolerance. These restorative strategies, including daytime napping, pacing of activities, and bodily adaptation, reflect attempts to maintain functional capacity and physical comfort despite fragmented or insufficient nocturnal sleep.

## DISCUSSION

In the present qualitative study, the main sleep problems reported by older adults were related to difficulty initiating and maintaining sleep, frequent nighttime awakenings, and shortened total sleep duration. These problems were closely linked not only to age-related physiological changes, but also to psychological distress, family-related worries, and environmental discomfort. Participants rarely reported receiving structured guidance about sleep from health care professionals or community health workers, and formal sleep management strategies were generally lacking. As a result, older adults relied on self-initiated approaches to cope with sleep problems, including spiritual practices, cognitive control of worries, behavioral adjustments, and daytime napping, with varying degrees of perceived effectiveness.

The findings of this study can be interpreted within a biopsychosocial perspective of sleep in later life. Supporting this view, previous work has shown that sleep is embedded within broader constellations of biological, psychological, and social characteristics, and that individuals may exhibit distinct sleep–biopsychosocial profiles rather than a single uniform pattern^(18)^. In our study, sleep quality was similarly not perceived solely as a biological or physiological process, but as something shaped by emotional experiences, social relationships, daily activities, and spiritual meaning. Integrating participants’ sleep experiences, coping strategies, and daytime consequences provided a comprehensive understanding of how older adults evaluate their sleep, respond to sleep difficulties, and appraise available support. The findings indicate the importance of sleep management strategies that address psychological well-being and social context alongside physiological aspects of ageing. Consistent with previous research, older adults commonly reported difficulty initiating and maintaining sleep, frequent nighttime awakenings, and shortened sleep duration; fragmented sleep was also frequently described and appeared to contribute to impaired daytime functioning^(19)^. Taken together, these findings suggest that the sleep problems identified in this study reflect both local contextual influences and broader age-related changes documented internationally.

The sleep experiences described in this study were deeply embedded in the Indonesian sociocultural context, particularly the Bangka Belitung Islands. In this setting, strong intergenerational family bonds, expectations of filial piety, and close-knit community life shaped how sleep problems were perceived and managed. These findings are supported by national research showing that insomnia among older adults is associated with limited family interaction and living alone following family separation due to children’s migration^(20)^. Previous research on family caregiving in Indonesia has shown that filial obligations strongly influence support for older adults; however, socioeconomic change and migration of adult children can disrupt these traditional networks, increasing vulnerability and potentially contributing to sleep disruption through isolation or changing family roles^(21)^. National survey data from older Indonesians have likewise demonstrated that insomnia symptoms are associated with social and sociodemographic factors, including low educational attainment, poor economic status, loneliness, and reduced life satisfaction, underscoring the important role of social and relational contexts in sleep^(22)^. Taken together, these findings suggest that sleep in later life in Indonesia is closely tied to family dynamics and social obligations, rather than being understood solely as an individual biomedical problem^(23)^. These findings suggest that sleep in later life in Indonesia is closely intertwined with family dynamics and social obligations, rather than being understood only as an individual health problem.

Environmental conditions also contributed to sleep disruption. Preferences regarding lighting, temperature, and noise varied widely, indicating that sleep quality is closely linked to older adults’ subjective perceptions of bedroom comfort. In Bangka Belitung, many participants lived in tropical coastal environments characterized by high temperatures, high humidity, and limited ventilation. They frequently described difficulty falling asleep and frequent nighttime awakenings during hot nights. These findings are consistent with field research among older adults in Shanghai showing that bedroom thermal conditions and ventilation are closely associated with objectively measured sleep quality^(24)^. In that study, higher bedroom air temperature and CO_2_ concentration were both negatively correlated with sleep outcomes. For every 1°C increase in air temperature, sleep efficiency decreased by about 0.7%, rapid eye movement (REM) sleep shortened by approximately 2.1 minutes, and time awake increased by about 2.3 minutes. Total sleep time also declined as CO_2_ increased, with an estimated reduction of around 11 minutes for each 100 ppm increase^(24)^. In addition to thermal conditions, participants frequently referred to light and noise intrusions from nearby houses, roads, or community activities, and earlier studies have similarly reported that environmental light exposure and nighttime noise are linked with sleep fragmentation and delayed sleep onset in older populations^(25)^.

A community-based study in Bengal reported that 87.5% of older adults experienced mosquito-related sleep disturbances at night, and that poor sleep quality was common, with 75% having PSQI scores ≥5 and a mean global PSQI of 8.33, indicating that insect exposure can be an important environmental source of sleep disruption in tropical settings^(26)^. Large cohort evidence also supports the contribution of light and noise to sleep disruption. In the Yiwu Elderly Cohort involving 2,978 adults aged 65 years and older, 39.7% were classified as having poor sleep quality (PSQI > 5), and both nighttime light and noise were significantly associated with poor sleep, with prevalence ratios of 1.14 and 1.15, respectively. Light from televisions or lamps left on in the room, light entering from outside, and noise from traffic and public places were associated with longer sleep latency, lower sleep efficiency, and more frequent sleep disturbances^(27)^. Together, these findings indicate that mosquitoes, light, and noise represent meaningful environmental contributors to the sleep problems described by participants in this study. Collectively, the convergence between participants’ accounts and previous evidence indicates that environmental factors such as heat, humidity, light, noise, and insects are not peripheral influences but are closely intertwined with how older adults experience and judge their sleep quality in everyday life. Because these environmental conditions differ substantially from those typically found in high-income countries, they also suggest that recommendations for sleep hygiene cannot simply be transferred from Western contexts but need to be adapted to Indonesian housing conditions and daily living environments.

The consequences of poor sleep were evident during daytime functioning, with participants reporting fatigue, sleepiness, reduced concentration, and decreased productivity, confirming that sleep problems affect not only nighttime rest but also daily activities and perceived health. This pattern is consistent with recent studies showing that poor sleep in older adults is associated with cognitive impairment, functional decline, and poorer quality of life^(28,29)^. Some participants described a perception of having adjusted to shorter sleep duration over time. This pattern may be interpreted as a form of response shift, in which expectations and internal standards regarding “adequate sleep” are recalibrated with ageing, rather than reflecting true improvement in sleep quantity or continuity^(30)^. Such reinterpretation aligns with theories of cognitive appraisal in aging, which suggest that age-related cognitive and motivational changes influence how older adults evaluate and make meaning of their experiences, including health-related symptoms, relative to younger counterparts. Older adults may thus normalize certain changes, reframe them as part of aging, or adapt coping strategies based on these appraisals, as shown in prior studies examining how older people interpret and manage stressful life challenges through appraisal and coping processes^(31)^. In response to these difficulties, older adults adopted multiple coping strategies, including pre-sleep routines, cognitive reframing of worries, emotional acceptance, and the use of spiritual practices. Similar qualitative studies have reported that older adults frequently rely on self-developed behavioral and cognitive strategies to manage insomnia symptoms^(32)^.

In the present study, these strategies were expressed not only through practical adjustments such as changing daily schedules or resting during the day, but also through broader forms of religious and spiritual coping. A scoping review has indicated that spiritual and religious interventions, are associated with improvements in sleep disturbances and insomnia outcomes, suggesting potential benefits of spiritual practices for sleep quality^(33)^. A randomized clinical trial similarly found that spiritual care programs incorporating religious support were associated with improved subjective sleep quality. Studies focusing on Qur’an recitation have shown reductions in anxiety and increases in feelings of calm, which may shape how individuals evaluate their sleep^(34)^. Research examining dhikr as a psychospiritual practice among older adults has also found that it fosters inner peace, lowers anxiety, and enhances emotional regulation, all of which are relevant to sleep experience^(35)^. Overall, these strands of evidence suggest that the religious and spiritual coping strategies described by participants align with wider literature showing that spirituality can support psychological comfort and more positive perceptions of sleep.

This study provides important insights into how older adults perceive sleep quality and cope with sleep disturbances in their daily lives. These findings are consistent with previous qualitative studies suggesting that sleep quality is shaped not only by physiological factors but also by social relationships, emotional well-being, environmental conditions, and cultural beliefs. The findings highlight that sleep disturbances are influenced not only by physical conditions, but also by psychological factors, family relationships, environmental comfort, and spiritual practices^(22)^. Understanding these experiences may help nurses, caregivers, and community health workers develop more individualized and culturally sensitive interventions to improve sleep quality among older adults. Greater attention to family support, sleep environment, and non-pharmacological coping strategies such as relaxation and spiritual practices may enhance sleep management and overall well-being in later life. These findings also support the role of community-based elderly services, such as Posyandu Lansia, in promoting healthy sleep and quality of life among older adults.

Several strengths and limitations should be acknowledged in this study. Sleep among older adults was explored through an in-depth qualitative approach, allowing rich descriptions of how sleep was experienced and managed in daily life. The inclusion of participants from both community settings and institutional care enhanced the diversity of perspectives. Methodological rigor was supported through systematic coding, triangulation, and the use of verbatim quotations. The study also highlighted the important roles of spirituality and family context in shaping sleep experiences and coping strategies. However, the relatively small, purposively selected sample from a single province, which did not represent all older adults in Bangka Belitung,, may limit the transferability of the findings and does not allow generalization to the entire older adult population of the province or to other cultural settings. As the data were based on self-reported experiences, recall bias and social desirability may have influenced the results. In addition, sleep quality was not objectively measured, so the findings reflect perceived rather than physiological sleep parameters. Despite these limitations, valuable insight was provided into sleep among older adults and a foundation was established for future research.

In summary, the findings of this study indicate that poor sleep in older adults is a complex phenomenon emerging from the interaction of biological ageing, psychological distress, social roles, environmental conditions, and spiritual meaning. Older adults do not experience sleep problems as isolated clinical symptoms, but as part of their broader life context, encompassing family relationships, health conditions, daily routines, and religious practices. At the same time, they rely largely on self-initiated strategies, with limited formal guidance from health services, which may leave some sleep problems insufficiently addressed. These results underscore the importance of adopting holistic, culturally sensitive approaches to sleep care for older adults in Indonesia, integrating psychological support, family and community involvement, environmental modification, and recognition of spiritual coping as legitimate resources. Future studies should examine how such multidimensional approaches can be translated into feasible interventions and how they may improve sleep quality and daytime functioning in later life.

## CONCLUSION

The findings of this study suggest that older adults in Bangka Belitung commonly experienced difficulty initiating and maintaining sleep, frequent night awakenings, and short sleep duration, while receiving limited guidance from health professionals. Many participants relied on self-initiated strategies such as spiritual practices, worry control, behavioral changes, and daytime napping, which were perceived as having varying levels of effectiveness and, in some cases, as being insufficient to address their sleep problems. These experiences highlight the importance of greater professional support and patient-centered sleep education to help older adults manage poor sleep quality and improve well-being.

## Data Availability

The entire dataset supporting the results of this study was published in the article itself.

## References

[1] He M, Yuan X, Zhao X, Liang H, Long Y, Zhang Y (2025). Sleep quality in older adults with cognitive frailty: current status and influencing factors. BMC Geriatr.

[2] Peng S, Chen Y, Li J, Wang Y, Liu X, Wang Y (2023). Correlation among sleep quality, physical frailty and cognitive function of the older adults in China: the mediating role. Front Public Health.

[3] Magnison-Benoit S, Snow C, Pun M, Gauba H, Berghmans S, Clarke A (2025). Age-related changes in sleep architecture: effects of body mass index, sex, and mental health in community-dwelling adults using at-home polysomnography. Sleep Med.

[4] Simon KC, Cadle C, Shuster AE, Malerba P (2025). Sleep across the lifespan: a neurobehavioral perspective. Curr Sleep Med Rep.

[5] Zamora AN, Roberts EF, Tellez-Rojo MM, Peterson KE, Torres-Olascoaga LA, Cantoral A (2025). Social drivers of sleep experiences: conversations with midlife working-class women from Mexico city. Research Directions: Sleep Psychology.

[6] Zhu C, Zhou L, Zhang X, Walsh CA (2023). Reciprocal effects between sleep quality and life satisfaction in older adults: the mediating role of health status. Healthcare (Basel).

[7] Li J, Vitiello MV, Gooneratne NS (2018). Sleep in normal aging. Sleep Med Clin.

[8] Bragazzi NL, Mutti C, Ratti PL, Sardella A, Quattropani M, Lodi R (2025). The complex interplay between sleep and healthy aging: a scoping review. Nat Sci Sleep.

[9] Savage RD, Hardacre K, Bashi AM, Bronskill SE, Faulkner C, Grieve J (2021). Perspectives on ageing: a qualitative study of the expectations, priorities, needs and values of older people from two Canadian provinces. Age Ageing.

[10] Aviisah MA, Haisma HH, Zotor FB, Vogt TC (2025). “Inner Peace is the Good Life”: conceptualizations of subjective well-being among older adults aged 60 and over in Rural Northern Ghana. J Happiness Stud.

[11] Gibson R, Lowe H, Korohina E, Rolleston A (2024). Māori perspectives on sleep and aging. Front Sleep.

[12] Gunawan J, Nazliansyah, Aungsuroch Y, Montayre J (2025). A qualitative study of coping strategies for loneliness among Indonesian older adults: implications for nursing practice. Int J Ment Health Nurs.

[13] Hidayat S, Kusnanto K, Hannan M (2021). Elderly sleep quality in crosscultural perspective. Journal of International Dental and Medical Research.

[14] Zhu W, Wang Y, Tang J, Wang F (2024). Sleep quality as a mediator between family function and life satisfaction among Chinese older adults in nursing home. BMC Geriatr.

[15] Oranga J, Matere A (2023). Qualitative research: essence, types and advantages. OAlib.

[16] Buysse DJ, Reynolds III CF, Monk TH, Berman SR, Kupfer DJ (1989). The Pittsburgh sleep quality index: a new instrument for psychiatric practice and research. Psychiatry Res.

[17] Braun V, Clarke V (2006). Using thematic analysis in psychology. Qual Res Psychol.

[18] Perrault A, Kebets V, Kuek N, Cross N, Tesfaye R, Pomares F (2024). A multidimensional investigation of sleep and biopsychosocial profiles with associated neural signatures [Internet].

[19] Gulia KK, Kumar VM (2018). Sleep disorders in the elderly: a growing challenge. Psychogeriatrics.

[20] Adawiyah AR, Djokosujono K, Sanga JL (2022). Sleep quality and affecting factors among elderly living in a nursing home at East Nusa Tenggara Indonesia. Indo J Public Health Nutr.

[21] Setiyani R, Windsor C, Douglas C (2019). Filial piety: from the perspective of indonesian young adults. Nurs Med J Nursing.

[22] Handajani YS, Schroeder-Butterfill E, Hogervorst E, Turana Y, Hengky A (2024). Insomnia in Indonesia older adults: the role of mental health, sociodemographic status, and physical function. Sleep Med Res.

[23] Budiman SS, Hasan F, Apriliyasari RW (2024). Comparative insomnia prevalence between geriatrics lived in urban and rural areas: a multicenter nationwide study analysis. BMC Public Health.

[24] Yan Y, Lan L, Zhang H, Sun Y, Fan X, Wyon DP (2022). Association of bedroom environment with the sleep quality of elderly subjects in summer: A field measurement in Shanghai, China. Build Environ.

[25] Mortazavi SS, Foroughan M, Hosseini SA, Nasiri E, Shahbazi F (2021). Negative factors affecting the sleep quality of the elderly in Iran: a systematic review. Arshiv-i Tavan’bakhshi.

[26] Inba RA, Sardar JC (2022). Sleep quality and its associated factors among elderly population in a rural area of West Bengal. Int J Community Med Public Health.

[27] Dai S, Wang W, Yang K, Li J, Duoliken H, Fang L (2025). Exposure to light and noise at night, and sleep quality in community-dwelling older adults: a cross-sectional study. Eur Geriatr Med.

[28] Aliño M, Molins F, Molins M, González L, Duque A, Mesa-Gresa P (2025). Better sleep, better minds: sleep as a key to cognitive performance and quality of life in aging [Internet].

[29] Künstler EC, Bublak P, Finke K, Koranyi N, Meinhard M, Schwab M (2023). The relationship between cognitive impairments and sleep quality measures in persistent insomnia disorder. Nat Sci Sleep.

[30] Hjort Telhede E (2025). Experiences of insomnia among older people living in nursing homes: a qualitative study. Int J Qual Stud Health Well-being.

[31] Young NA, Minton AR, Mikels JA (2021). The appraisal approach to aging and emotion: an integrative theoretical framework. Dev Rev.

[32] McLaren DM, Evans J, Baylan S, Smith S, Gardani M (2023). The effectiveness of the behavioural components of cognitive behavioural therapy for insomnia in older adults: a systematic review. J Sleep Res.

[33] De Diego-Cordero R, Acevedo-Aguilera R, Vega-Escaño J, Lucchetti G (2022). The use of spiritual and religious interventions for the treatment for insomnia: a scoping review. J Relig Health.

[34] Yousofvand V, Torabi M, Oshvandi K, Kazemi S, Khazaei S, Khazaei M (2023). Impact of a spiritual care program on the sleep quality and spiritual health of Muslim stroke patients: a randomized controlled trial. Complement Ther Med.

[35] Sukandar W (2025). Dhikr as a psychospiritual therapy for the elderly: a phenomenological study at the Ibnu Masud Elderly Boarding School, Segamat, Johor, Malaysia. Jurnal Kajian Dan Pengembangan Umat.

