# Supplementary material for: Experiences and perceptions of sleep quality among older adults in Bangka Belitung, Indonesia: a qualitative study
Source: Rev Esc Enferm USP. 2026 Sep 7;60:e20260008. doi: 10.1590/1980-220X-REEUSP-2026-0008en (PMC13557006; doi:10.1590/1980-220X-REEUSP-2026-0008en)
Supplement: Translation

# Translation: Experiencias y percepciones sobre la calidad del sueño entre adultos mayores en Bangka Belitung, Indonesia: un estudio cualitativo

**DOI:** 10.1590/1980-220X-REEUSP-2026-0008es

**Authors:** Indah Permata Sari, Tita Hariyanti, Retno Lestrari, Respati Suryanto Dradjat

**Affiliations:** 1 Universitas Brawijaya, Faculty of Medicine, Doctoral Program in Medical Science, Malang, Indonesia.; 2 Institut Citra Internasional, Faculty of Nursing, Department of Nursing, Pangkalpinang, Indonesia.; 3 Universitas Brawijaya, Faculty of Medicine, Department of Public Health, Malang, Indonesia.; 4 Universitas Brawijaya, Faculty of Health Sciences, Department of Nursing, Malang, Indonesia.; 5 Universitas Brawijaya, Faculty of Medicine, Department of Orthopaedic and Traumatology, Malang, Indonesia.; 6 Dr. Saiful Anwar Regional General Hospital, Department of Orthopaedic and Traumatology, Malang, Indonesia.

**Keywords:** Trastornos del Sueño-Vigilia, Anciano, Calidad Del Sueño, Investigación Cualitativa, Habilidades de Afrontamiento

## Abstract

**Objetivo:**

Explorar las percepciones y experiencias de los ancianos sobre el sueño y las estrategias que utilizan para afrontar su mala calidad.

**Método:**

Se realizó un estudio descriptivo cualitativo con 27 ancianos seleccionados intencionadamente de entornos comunitarios e institucionales en Bangka Belitung. Los datos se recopilaron entre junio y agosto de 2025 mediante entrevistas en profundidad y se analizaron temáticamente con triangulación, verificación por parte de los participantes y cumplimiento de las directrices COREQ.

**Resultados:**

La mayoría de los participantes eran mujeres de entre 60 y 74 años, muchas de ellas viudas y con enfermedades crónicas. Surgieron dos temas principales: las malas experiencias de sueño y las estrategias para afrontarlas. Entre los problemas más comunes se encontraban la dificultad para conciliar y mantener el sueño, los despertares nocturnos, el despertar temprano y la fatiga diurna. Los trastornos del sueño se relacionaron con el distrés psicológico y la incomodidad ambiental. Las estrategias de afrontamiento incluyeron cambios de comportamiento, prácticas espirituales, siestas diurnas y ajustes en las actividades.

**Conclusión:**

Los resultados de este estudio sugieren que el sueño entre los ancianos participantes en Bangka Belitung se percibe como multidimensional, influenciado por factores fisiológicos, psicológicos, ambientales y espirituales, lo que subraya la necesidad de un apoyo comunitario y culturalmente sensible que vaya más allá de las estrategias autoiniciadas.

## INTRODUCCIÓN

La calidad del sueño es un componente central de la salud y el bienestar en los adultos mayores, ya que influye en el funcionamiento físico, la cognición, la regulación emocional y la calidad de vida en general^(1,2)^. El envejecimiento va acompañado de cambios fisiológicos en la arquitectura del sueño, tales como una menor duración del sueño, un aumento de los despertares nocturnos y una reducción del sueño profundo^(3)^. Sin embargo, el sueño en la tercera edad no está determinado únicamente por la biología; los roles sociales, las rutinas diarias, las condiciones de vida y las normas culturales también influyen en cómo las personas mayores experimentan e interpretan el sueño^(4,5,6)^. En muchas comunidades, los problemas de sueño en las personas mayores se normalizan como una parte natural del envejecimiento y siguen sin reportarse en su totalidad. Como resultado, el significado subjetivo de un sueño “bueno” o “malo” entre las personas mayores a menudo difiere de las definiciones clínicas.

La comprensión de la calidad del sueño en la vejez puede abordarse no solo a través de los síntomas y los indicadores clínicos, sino también a través de la forma en que las personas mayores conceptualizan y le dan sentido a su sueño^(7)^. Si bien muchos estudios previos se han centrado principalmente en evaluaciones cuantitativas y resultados medibles, como las puntuaciones del Índice de Calidad del Sueño de Pittsburgh (PSQI), la duración del sueño y la eficiencia del sueño, son menos los que han examinado los significados subjetivos y las interpretaciones asociadas al sueño en la vejez^(8)^. Los enfoques cualitativos ofrecen una valiosa perspectiva sobre cómo las expectativas respecto al envejecimiento, las condiciones de salud, los estados emocionales, la interacción familiar y los contextos ambientales pueden entrecruzarse con las experiencias relacionadas con el sueño^(9)^. Dichas perspectivas son particularmente relevantes en entornos donde los contextos sociales y culturales pueden influir en las creencias sobre el sueño y el envejecimiento. Por lo tanto, examinar estas dimensiones puede contribuir a una comprensión teórica más contextualizada del sueño en la tercera edad^(10,11)^.

Bangka Belitung, una provincia insular de Indonesia, representa un contexto en el que las condiciones socioculturales pueden moldear las experiencias relacionadas con el sueño en la vejez. Los patrones de actividad diaria, las formas de vida comunitaria, las prácticas religiosas y los medios de subsistencia propios de una isla pueden estar relacionados con los horarios de sueño, las oportunidades de descanso y los despertares nocturnos. Además, la soledad se ha convertido en un tema importante que afecta el bienestar de las personas mayores en todo el mundo y puede influir en las experiencias de sueño, especialmente a medida que los cambios culturales y la urbanización aumentan la vulnerabilidad al aislamiento social^(12)^. Aunque los problemas de sueño entre las personas de la tercera edad se han estudiado ampliamente, la mayoría de las investigaciones anteriores se han centrado principalmente en indicadores cuantitativos como la duración del sueño, la eficiencia del sueño y los puntajes del PSQI. La evidencia previa también ha demostrado que la calidad del sueño en las personas de la tercera edad se ve influenciada por múltiples factores sociales y ambientales, entre ellos el apoyo familiar, las relaciones sociales y las condiciones de vida^(13)^. Sin embargo, las investigaciones que examinan cómo las personas de la tercera edad perciben e interpretan subjetivamente sus experiencias de sueño dentro de su contexto sociocultural siguen siendo limitadas. Los estudios han destacado la importancia de las perspectivas culturales para comprender el sueño en las personas mayores; sin embargo, la evidencia cualitativa que explore el papel de las relaciones familiares, la espiritualidad, las condiciones ambientales y las creencias culturales en la configuración de las experiencias de sueño sigue siendo escasa, particularmente en el contexto indonesio. Además, se sabe poco sobre cómo las personas mayores manejan la falta de sueño mediante estrategias de afrontamiento propias, integradas en las prácticas socioculturales locales. Esta brecha limita una comprensión integral de cómo el sueño se integra en la vida cotidiana y restringe el desarrollo de intervenciones culturalmente sensibles^(13,14)^. Por lo tanto, este estudio tiene como objetivo explorar las percepciones y experiencias de sueño de las personas mayores, así como las estrategias que utilizan para hacer frente a la mala calidad del sueño entre las personas mayores en Bangka Belitung. Se espera que estas experiencias de sueño y estrategias de afrontamiento estén estrechamente vinculadas con las normas socioculturales, las relaciones familiares, las condiciones ambientales y las prácticas espirituales.

## MÉTODO

### Diseño del Estudio

Este estudio empleó un diseño cualitativo descriptivo para explorar los patrones y la calidad del sueño entre las personas mayores. Este enfoque permite una descripción integral de un fenómeno tal como lo narran directamente los participantes, con una interpretación teórica previa mínima. La investigación cualitativa hace hincapié en la comprensión de las experiencias humanas y los fenómenos situados socialmente a través de relatos ricos y no numéricos; por lo tanto, se consideró apropiada para el presente estudio^(15)^.

### Población y Ubicación

La población objetivo estuvo compuesta por adultos mayores que residen en la provincia de Bangka Belitung, Indonesia. La provincia cuenta con aproximadamente 50 centros comunitarios de servicios de salud para adultos mayores y 2 centros residenciales de atención a adultos mayores. Los participantes fueron reclutados en sitios seleccionados en función de la accesibilidad, la disponibilidad de los participantes y la disposición del personal local a apoyar el estudio, dentro de la provincia entre junio y agosto de 2025. El estudio se centró en cómo las personas mayores, tanto las que vivían en la comunidad como las institucionalizadas, experimentaban, interpretaban y manejaban el sueño en estos entornos.

### Criterios de Inclusión y Exclusión

Se incluyó en el estudio a los participantes que tenían 60 años o más, informaban tener problemas de sueño —como dificultad para conciliar el sueño, despertares nocturnos frecuentes, despertarse muy temprano por la mañana, duración corta del sueño, sueño no reparador o disfunción diurna relacionada con la falta de sueño—, residían en los entornos seleccionados para el estudio, podían comunicarse de manera efectiva durante la entrevista y proporcionaron su consentimiento informado por escrito. Se excluyó a los participantes si padecían una enfermedad grave, limitaciones físicas importantes o trastornos psicológicos graves que pudieran interferir con su participación, o si no residían en los lugares designados para el estudio. Participaron en el estudio un total de 27 adultos mayores, incluidos 22 participantes que vivían en la comunidad a través de puestos de servicios de salud integrados para adultos mayores y 5 participantes que residían en centros de atención para adultos mayores. La mayoría de los participantes que vivían en la comunidad convivían con familiares, mientras que los participantes en centros de atención residencial compartían espacios de vida con otros residentes sin vínculos familiares. Se consideró que estas diferentes condiciones de vida eran importantes para comprender las variaciones en las experiencias de sueño, el apoyo emocional y la comodidad ambiental.

### Muestra y Reclutamiento

Se utilizó un muestreo intencional para reclutar a adultos mayores que se consideraban capaces de proporcionar información rica y detallada sobre sus experiencias de sueño. Para captar perspectivas diversas, se reclutó a participantes de diferentes distritos de Bangka Belitung y tanto del ámbito comunitario como de centros de atención residencial. El reclutamiento se llevó a cabo en coordinación con el personal de los puestos de salud integrados para personas mayores y de los centros de atención residencial, quienes ayudaron a identificar a los adultos mayores elegibles según los criterios de inclusión. La selección final de los participantes la realizó el investigador. Las entrevistas continuaron hasta alcanzar la saturación de datos, es decir, cuando ya no surgieron nuevos temas ni información relevante de las entrevistas posteriores. Participaron en el estudio un total de 27 adultos mayores. Todos los participantes que dieron su consentimiento informado completaron la entrevista, y no hubo desistimientos durante la recolección de datos.

### Procedimiento de Recolección de Datos

Los datos se recolectaron mediante entrevistas individuales, presenciales y en profundidad, que contenían 32 preguntas abiertas destinadas a explorar las experiencias de sueño entre las personas mayores. Las entrevistas fueron realizadas por la investigadora principal, quien cuenta con formación en enfermería y experiencia en entrevistas cualitativas con personas mayores. La guía de entrevista se desarrolló utilizando el marco del PSQI, introducido originalmente por Buysse et al.^(16)^, como base conceptual y no como un instrumento cuantitativo estandarizado. La guía se organizó en torno a siete dimensiones derivadas del PSQI y se amplió para reflejar aspectos culturalmente relevantes del sueño entre las personas mayores en Indonesia, incluyendo aspectos psicológicos y emocionales, patrones de sueño, comportamientos y estilo de vida, estado de salud y condiciones físicas, perturbaciones externas, características del entorno físico para dormir y disfunción diurna. Se incluyeron preguntas de sondeo adicionales para explorar las relaciones familiares, las normas socioculturales y las prácticas espirituales relacionadas con el sueño.

Para garantizar la validez de contenido y la relevancia contextual, la guía de entrevista fue sometida a la evaluación de expertos, en la que participaron especialistas en enfermería, profesionales de la atención a personas mayores y un experto en psicología. Se realizaron revisiones basadas en sus comentarios sobre la claridad, la relevancia y la adecuación cultural de las preguntas. Las entrevistas se realizaron en los hogares de los participantes, en el caso de las personas mayores que vivían en la comunidad, y en habitaciones privadas dentro de centros de atención residencial, en el caso de los participantes institucionalizados, para garantizar la confidencialidad y la comodidad. Cada entrevista duró aproximadamente entre 45 y 60 minutos, se grabó en audio con el permiso del participante y se complementó con notas de campo que documentaban expresiones no verbales y observaciones contextuales. Durante las entrevistas no estuvo presente ninguna otra persona además del participante y el entrevistador. La guía completa de la entrevista se presenta en la Tabla 1.

**Tabla 1:** Guía de entrevista para analizar la calidad del sueño en personas mayores – Bangka Belitung, Indonesia, 2024.

N.º	Pregunta de la entrevista
**Sección 1. Información general**	
1	¿Podrías contarme un poco sobre tu vida cotidiana y tus actividades habituales?
2	¿Vives solo, con tu pareja, con tus hijos o con otros familiares?
3	¿Padece alguna enfermedad crónica o toma algún medicamento de forma habitual?
**Sección 2. Hábitos y patrones de sueño**	
4	¿A qué hora suele acostarse por la noche? ¿Tiene un horario fijo para acostarse?
5	¿A qué hora sueles levantarte por la mañana? ¿Te despiertas de forma espontánea o con la ayuda de otras personas o de un despertador?
6	¿Sueles echarte la siesta durante el día? En caso afirmativo, ¿cuánto tiempo sueles dormir?
7	¿Cuál es tu rutina habitual antes de acostarte? ¿Qué sueles hacer antes de irte a dormir?
8	¿Han cambiado tus hábitos de sueño en comparación con hace unos años? ¿Qué ha cambiado y por qué?
**Sección 3. Entorno para dormir**	
9	¿Duermes en una habitación individual, con tu pareja o con otros miembros de la familia? ¿Influye esto en tu sueño?
10	¿Te parecen cómodos el colchón, la almohada y la manta? ¿Sientes dolor o rigidez después de dormir?
11	¿Cómo es la iluminación de tu dormitorio cuando duermes: oscura, tenue o brillante?
12	¿Los ruidos del exterior o del interior de la casa te perturban el sueño?
13	¿Cómo es la temperatura de la habitación por la noche: hace calor, frío o es agradable? ¿Utilizas un ventilador o aire acondicionado?
14	¿Cómo es la calidad del aire en tu habitación: húmedo, viciado o fresco? ¿Abres la ventana o utilizas algún aparato?
15	En tu opinión, ¿hay algo en tu dormitorio que se podría mejorar para ayudarte a dormir mejor?
**Sección 4. Estilo de vida y factores psicológicos**	
16	¿Realizas alguna actividad física o ejercicio? ¿De qué tipo y cuándo?
17	¿Consumes cafeína (café, té, bebidas energéticas) por la tarde o por la noche?
18	¿Fumas o bebes alcohol? En caso afirmativo, ¿lo haces cerca de la hora de acostarte?
19	¿Te sientes ansioso, triste o te dan muchas vueltas a la cabeza antes de dormir? ¿Cómo afecta esto a tu sueño?
20	¿Alguna vez te has sentido solo o inquieto por la noche? ¿Cómo lo afrontas?
21	¿Utilizas pastillas para dormir, suplementos o remedios tradicionales que te ayuden a conciliar el sueño? En caso afirmativo, ¿qué eficacia tienen?
**Sección 5. Percepciones e impacto del sueño**	
22	¿Consideras que duermes lo suficiente y que tu sueño es de buena calidad?
23	¿Te sientes a menudo cansado, con sueño o tienes dificultades para concentrarte durante el día?
24	¿Qué significa para ti “dormir bien”? ¿Qué esperas de una buena noche de sueño?
25	¿Qué sueles hacer cuando no consigues conciliar el sueño? ¿Qué es lo que más te ayuda?
26	¿Alguna vez has consultado a un profesional sanitario por problemas de sueño? ¿Qué consejos te dieron?
27	¿Crees que dormir bien es importante para tu salud? ¿Por qué?
**Sección 6. Aspectos espirituales, sociales y relacionados con el envejecimiento**	
28	¿Tienes alguna práctica espiritual antes de dormir (oración, dhikr, meditación)? ¿Te ayudan a dormir?
29	¿Ha cambiado tu sueño desde que te has hecho mayor? ¿Cuáles son las principales diferencias?
30	¿Afecta a tus hábitos de sueño el hecho de vivir con hijos o nietos? ¿De qué manera?
**Preguntas finales**	
31	¿Hay algo más que le gustaría compartir sobre su experiencia con el sueño que no hayamos comentado?
32	En su opinión, ¿qué es lo más importante para que las personas mayores duerman bien por la noche?

### Análisis de Datos

Los datos se analizaron mediante un análisis temático siguiendo el marco propuesto por Braun y Clarke^(17)^. Las entrevistas grabadas en audio se transcribieron textualmente y se leyeron repetidamente para familiarizarnos con los datos. Las transcripciones y las notas de campo que documentaban las expresiones no verbales se importaron al software NVivo 12 para facilitar la gestión de los datos. Se identificaron segmentos significativos de texto, se condensaron en unidades de significado, se codificaron y, posteriormente, se agruparon en categorías y temas generales que reflejaban patrones recurrentes en los relatos de los participantes. El análisis fue iterativo e implicó una comparación constante entre los datos brutos, los códigos, las categorías y los temas para mantener la fidelidad a los relatos de los participantes. La triangulación se llevó a cabo utilizando las transcripciones de las entrevistas, las notas de campo y las notas reflexivas, mientras que la verificación por parte de los participantes se realizó con cinco participantes procedentes tanto del ámbito comunitario como del institucional para confirmar la credibilidad y la coherencia de las interpretaciones. Se mantuvieron notas reflexivas a lo largo de todo el proceso para mejorar la conciencia sobre los supuestos de los investigadores y minimizar el sesgo.

### Aspectos Éticos

Se obtuvo la autorización ética para este estudio por parte del Comité de Ética de la Universitas Brawijaya (n.º de autorización 199/EC/KEPK-S3/07/2025). Se obtuvo el consentimiento informado por escrito de todos los participantes tras explicarles los objetivos, los procedimientos, los posibles riesgos y los beneficios del estudio. Se garantizaron la confidencialidad y el anonimato mediante el uso de códigos de identificación y el almacenamiento seguro de los materiales de investigación. La participación fue voluntaria, y se informó a los participantes de su derecho a retirarse en cualquier momento sin consecuencias negativas.

## RESULTADOS

### Características de los Participantes

Las características demográficas de los participantes se presentan en la Tabla 2. En el estudio participaron un total de 27 personas mayores, de las cuales 22 procedían de entornos comunitarios y 5 de centros residenciales. Los participantes que vivían en la comunidad fueron seleccionados en cuatro regencias —a saber, Bangka (n = 6), Bangka Occidental (n = 5), Bangka Central (n = 5) y Bangka Meridional (n = 4)—, así como en un municipio, Pangkalpinang (n = 2), mientras que los 5 participantes restantes procedían de dos centros residenciales de atención a personas mayores. La mayoría de los participantes pertenecían al grupo de edad de la tercera edad más joven y la mayoría eran mujeres. El nivel educativo era, en general, bajo o moderado, y la mayoría de los participantes había completado la educación primaria o secundaria. Más de la mitad de los participantes eran viudos, y la mayoría no tenía un empleo formal, identificándose muchos de ellos como amas de casa o jubilados. Una proporción considerable de participantes declaró padecer una o más enfermedades crónicas. Los problemas de salud más frecuentes incluían la hipertensión y la hipercolesterolemia, seguidos de la diabetes y los problemas respiratorios. Solo un pequeño número de participantes declaró no padecer ninguna enfermedad crónica.

**Tabla 2:** Características demográficas de los participantes en el estudio cualitativo – Bangka Belitung, Indonesia, 2025 (n = 27).

Variable	Categoría	n (%)
Edad	60–64 años	11 (40,7)
	65–69 años	7 (25,9)
	70–74 años	4 (14,8)
	≥ 75 años	5 (18,5)
Sexo	Hombres	3 (11,1)
	Mujer	24 (88,9)
Nivel educativo más alto	Educación primaria	10 (37,0)
	Educación secundaria de primer ciclo	6 (22,2)
	Bachillerato	9 (33,3)
	Diploma	2 (7,4)
Estado civil	Casado	12 (44,4)
	Viudo	15 (55,6)
Ocupación actual	Desempleada/ama de casa	15 (55,6)
	Trabajadora ocasional	1 (3,7)
	Comerciante/autónomo	5 (18,5)
	Agricultor	1 (3,7)
	Jubilado	5 (18,5)
Antecedentes médicos*	Sin enfermedades crónicas	9 (23,7)
	Hipertensión	10 (26,3)
	Diabetes mellitus	4 (10,5)
	Hipercolesterolemia	8 (21,1)
	Gota	2 (5,3)
	Gastritis	2 (5,3)
	Enfermedad respiratoria/asma	3 (7,9)

*Los participantes pueden indicar más de una enfermedad crónica; por lo tanto, el número total de respuestas sobre el historial médico fue de 38, y los porcentajes se calcularon basándose en n = 38.

El análisis cualitativo generó dos temas principales con varios subtemas relacionados. El primer tema reflejaba experiencias de mala calidad del sueño, y el segundo describía las estrategias utilizadas para gestionar los problemas de sueño. Estos temas se derivaron del proceso de codificación y categorización durante el análisis de los datos. A continuación se describen los temas y subtemas, con citas ilustrativas de los participantes que se muestran en las Tablas 3 y 4.

**Tabla 3:** Tema 1: experiencias de mala calidad del sueño entre las personas mayores – Bangka Belitung, Indonesia, 2024.

Cita		Subtema		Categoría
“Normalmente me acuesto a las 10 o a las 11 y media”.		Hora habitual de acostarse		Horario de sueño
“A veces ya estoy dormido antes de las 10”.		Hora de acostarse variable		Horario de sueño
“Me despierto sobre las 3 de la madrugada, de forma espontánea”.		Despertares tempranos por la mañana		Fragmentación del sueño
“Me despierto entre las 2 y las 3 de la madrugada y luego no consigo volver a dormirme”.		Dificultad para volver a conciliar el sueño		Insomnio de mantenimiento del sueño
“Desde que cumplí los cincuenta, me cuesta conciliar el sueño temprano”.		Cambio en los patrones de sueño con la edad		Cambio en los patrones de sueño
“A veces me despierto para rezar el tahajud”.		Despertar nocturno por motivos religiosos		Despertares nocturnos
“A menudo me despierto porque el nieto está llorando”.		Despertado por otras personas		Perturbaciones sociales o ambientales
“Si hay el más mínimo ruido, me despierto”.		Sensibilidad al ruido		Factores sensoriales
“Si hace calor, no puedo dormir”.		Intolerancia al calor		Factor ambiental
“Cuando hace frío, me despierto a menudo”.		Sensibilidad al frío		Factor ambiental
“A veces, dos veces por semana, me cuesta conciliar el sueño”.		Insomnio episódico		Trastorno del sueño
“A menudo tengo sueño durante el día”.		Somnolencia diurna		Consecuencias diurnas
“Siento que me tiembla el cuerpo cuando me falta sueño”.		Inestabilidad física		Consecuencias durante el día
“Cuando me despierto, me duele la espalda”.		Dolor matutino		Síntoma físico
“A veces no puedo concentrarme”.		Disminución de la concentración		Consecuencia cognitiva
“Durante el día me da pereza trabajar”.		Menor productividad		Consecuencia funcional
“Me enfado con facilidad”.		Irritabilidad		Consecuencia emocional
“Me preocupa que mi nieto esté enfermo”.		Preocupación por la familia		Factor psicológico
“Me siento triste al estar sola por la noche”.		Soledad		Factor emocional
“Tengo muchas cosas en la cabeza… y por eso no puedo dormir”.		Pensar demasiado/rumiación		Factor psicológico
“Me da miedo la oscuridad”.		Miedo a la oscuridad		Factor psicológico
“Me da miedo estar solo en casa”.		Miedo a estar solo		Inseguridad percibida
“Echo de menos a mi cónyuge, que ha fallecido”.		Duelo		Factor emocional
“No puedo dormir si está oscuro”.		Intolerancia a la luz		Entorno para dormir
“La luz tiene que ser tenue”.		Preferencia lumínica		Entorno para dormir
“Hay mucho ruido, pero ya me he acostumbrado”.		Exposición al ruido		Entorno para dormir
“El colchón es cómodo”.		Percepción del colchón		Entorno para dormir
“La habitación es pequeña, pero cómoda”.		Comodidad de la habitación		Entorno para dormir
“Dormir te da energía”.		Significado del sueño		Importancia percibida
“Dormir es importante para la salud”.		Sueño y salud		Importancia percibida

**Tabla 4:** Tema 2: estrategias utilizadas para gestionar los problemas de sueño entre las personas mayores – Bangka Belitung, Indonesia, 2025.

Cita		Subtema		Categoría
“Rezo antes de irme a dormir”.		Ritual antes de acostarse		Superación espiritual
“Recitar el Yasin me da sueño por sí solo”.		Relajación religiosa		Afrontamiento espiritual
“Si no puedo dormir, leo el Corán”.		Estrategia religiosa		Afrontamiento espiritual
“Bebo leche”.		Consumo antes de dormir		Estrategia conductual
“A veces veo la tele”.		Ver la tele antes de dormir		Hábitos con los medios
“Reviso el grupo de WhatsApp en mi móvil”.		Uso del móvil		Hábitos de consumo de medios
“Primero me acuesto y me relajo”.		Relajación antes de dormir		Preparación conductual
“Primero ordeno la casa”.		Actividad doméstica ligera		Estrategias de afrontamiento conductuales
“No tengo ninguna rutina especial”.		Falta de rutina para dormir		Comportamiento durante el sueño
“Tomo mi medicación para la tensión arterial”.		Uso de medicación		Estrategia terapéutica
“Me gusta sentarme tranquilamente primero”.		Sentarse en silencio		Relajación
“Si estoy cansado, solo entonces puedo echar una siesta”.		Siesta compensatoria		Estrategia de recuperación
“La siesta diurna es imprescindible… si no, me pongo de mal humor”.		Siesta programada		Estrategia de recuperación
“Intento controlar mis pensamientos”.		Control de los pensamientos		Afrontamiento cognitivo
“No te lleves demasiados pensamientos a la cama”.		Reformulación cognitiva		Estrategia de afrontamiento
“El dhikr me tranquiliza”.		Calma emocional		Afrontamiento espiritual

### Principales Conclusiones del Estudio

El análisis cualitativo generó dos temas principales con varios subtemas relacionados. El primer tema reflejaba experiencias de mala calidad del sueño, y el segundo describía las estrategias utilizadas para gestionar los problemas de sueño. Estos temas se derivaron del proceso de codificación y categorización durante el análisis de los datos. A continuación, se describen los temas y subtemas, con citas ilustrativas de los participantes que se muestran en las Tablas 3 y 4.

### Experiencias de Mala Calidad del Sueño en Adultos Mayores

#### Patrones de Sueño en Adultos Mayores

En general, los patrones de sueño de los adultos mayores se caracterizaron por acostarse relativamente tarde y una duración corta del sueño nocturno. Muchos adultos mayores informaron que se acostaban entre las 22:00 y las 23:00, y algunos solo lograban conciliar el sueño después de la medianoche. Un participante de Belinyu describió que “a veces es después de las once cuando finalmente puedo dormir… a veces incluso en medio de la noche”. Otro participante explicó que esta tendencia a acostarse tarde solo había surgido en los últimos años, y señaló que “desde que cumplí los cincuenta me ha resultado difícil conciliar el sueño temprano”. La mayoría de los participantes también informaron de que se despertaban muy temprano por la mañana, por lo general entre las 02:00 y las 05:00, a menudo sin ningún desencadenante específico. Varias personas mayores afirmaron que hacía mucho tiempo que se habían acostumbrado a despertarse muy temprano. Como expresó un participante: “A las cuatro ya estoy despierto y ya no puedo volver a dormirme”. Entre quienes residían en hogares de atención, despertarse muy temprano se había convertido en parte de su rutina diaria; un participante afirmó: “A las dos ya estoy despierto… simplemente me quedo sentado hasta la mañana”. Al reflexionar sobre etapas anteriores de su vida, los participantes percibieron cambios claros en su sueño. Muchos afirmaron que dormían mucho más profundamente cuando eran más jóvenes. Una mujer de edad avanzada señaló: “Cuando era joven dormía bien… ahora a menudo me despierto en medio de la noche”. En general, los participantes interpretaron estos cambios como una parte natural del proceso de envejecimiento.

### Fragmentación del Sueño y Despertares Nocturnos

La mayoría de las personas mayores experimentaban un sueño fragmentado, caracterizado por despertares frecuentes durante la noche y la incapacidad de volver a conciliar el sueño después. Varias personas mayores informaron que se despertaban para orinar, por dolores corporales o por pensamientos intrusivos. Un participante explicó: “A veces me despierto a la una, luego otra vez a las dos… es difícil volver a conciliar el sueño hasta el amanecer”. Aunque muchas personas mayores percibían esta situación como molesta, también describieron que habían aprendido gradualmente a aceptarla y a adaptarse a ella. Entre quienes vivían en centros de atención social, también se reportó con frecuencia el sueño fragmentado. Un participante afirmó que, después de despertarse a las dos de la mañana, no podía volver a conciliar el sueño: “No sé… simplemente me quedo en la cama hasta la mañana”. Algunos participantes se despertaban para realizar las oraciones nocturnas (tahajud), pero no siempre lograban volver a dormir después. Como describió un participante: “A veces me despierto a las tres y media para el tahajud… pero luego me cuesta volver a dormir”. La dificultad para volver a dormir a menudo provocaba cansancio y fatiga durante el día. No obstante, los participantes informaron que seguían realizando sus actividades diarias, como trabajar, preparar la comida o vender productos.

### Factores Psicológicos Que Contribuyen a los Trastornos del Sueño

Los factores psicológicos se revelaron como uno de los factores más importantes que contribuyen a los trastornos del sueño. Muchos participantes señalaron que las preocupaciones y los pensamientos intrusivos influían enormemente en su capacidad para dormir. Un participante que vivía en un centro de atención social, hablando con tono triste, afirmó: “A veces me siento triste… Pienso demasiado… Extraño a mis hijos”. Otro participante expresó profunda preocupación por un nieto con una enfermedad cardíaca, diciendo: “No dejo de pensar en mi nieto… Tengo miedo de que el tratamiento en Yakarta no tenga éxito”.

Varias personas mayores experimentaban un estrés considerable relacionado con la familia. Una abuela de Belinyu compartió su experiencia de haber sido rechazada por su nuera cuando deseaba vivir con su hijo, diciendo: “No es que no esté triste… pero mi hijo le tiene miedo a su esposa”. Tales experiencias intensificaban la carga emocional que vivían las personas mayores y tenían un impacto directo en la calidad de su sueño. La influencia del malestar psicológico se reflejó claramente en la forma en que los participantes describían su proceso de sueño. Algunos participantes explicaron que cuando tenían “demasiadas cosas en la cabeza, simplemente no podía dormir”, mientras que otros informaron que se acostaban durante mucho tiempo y, aun así, no lograban cerrar los ojos. Estas condiciones mentales y emocionales fueron los principales desencadenantes de las dificultades para dormir entre muchos participantes. Diversas experiencias psicológicas que afectaban el sueño, como la ansiedad, la rumiación excesiva y la soledad, tal como se resume en la Tabla 3, ilustraron la relación entre los factores psicológicos y la calidad del sueño entre las personas mayores.

### Factores Ambientales Que Contribuyen a un Sueño Deficiente

Las condiciones ambientales del dormitorio influían de manera diferente en el sueño según los participantes. Las preferencias con respecto a la iluminación variaban considerablemente. Algunos participantes afirmaron que solo podían dormir con la luz totalmente encendida, mientras que otros señalaron que no podían dormir a menos que la habitación estuviera completamente a oscuras. Un adulto mayor dijo: “Por lo general está oscuro… si hay luz, no puedo dormir”. Por el contrario, otro participante afirmó: “Debe haber una luz… si está oscuro, me cuesta respirar”. La temperatura de la habitación también se describió como un factor importante. Muchos participantes preferían los ventiladores eléctricos y evitaban los aires acondicionados porque sentían incomodidad física al exponerse al aire frío. Un participante explicó: “Cuando uso el aire acondicionado me duelen los huesos… pero con el ventilador no tengo problema”. En general, la mayoría de los participantes no percibía el ruido ambiental como una molestia importante. Varios de ellos incluso señalaron que, una vez que se habían quedado dormidos, ya no notaban los sonidos del exterior, como comentó un participante: “Una vez que estoy dormido… duermo… ya no oigo nada”. La mayoría de los participantes consideraban que sus colchones y almohadas eran cómodos. Sin embargo, algunas personas mayores informaron sentir dolor o rigidez al despertarse, especialmente entre quienes realizaban trabajos físicamente exigentes o tenían problemas articulares.

### Consecuencias Diurnas de la Falta de Sueño

La falta de sueño nocturno provocó una serie de consecuencias diurnas entre los participantes. Varios participantes informaron sentir somnolencia diurna excesiva, fatiga y disminución de la concentración. Un participante que trabajaba en un centro de atención social afirmó: “Durante el día a menudo me siento somnoliento… cansado… pero no puedo dormir la siesta porque tengo que trabajar”. Esta situación sugiere que la oportunidad limitada de descansar durante el día puede agravar los efectos de un sueño nocturno inadecuado. Por el contrario, algunas personas mayores describieron que sus cuerpos ya se habían adaptado a una duración más corta del sueño nocturno. Un participante explicó: “Gracias a Dios es suficiente… por lo general me despierto a las cuatro y no siento mareos”. Esto sugiere una variación individual considerable en la tolerancia a la duración del sueño. Varios participantes también recurrían a las siestas diurnas como estrategia compensatoria. Las siestas cortas, de aproximadamente 30 a 60 minutos, se consideraban útiles para recuperar energía y reducir la fatiga a lo largo del día. Esta práctica era especialmente común entre los participantes que no podían volver a conciliar el sueño después de despertarse durante la noche.

### Significado e Importancia Percibidos del Sueño

La mayoría de los participantes percibían el sueño como un elemento esencial tanto para la salud física como emocional. El sueño se percibía como un medio para recuperar energía después de las actividades diarias, tal como lo refleja la declaración de un adulto mayor que dijo: “El sueño repone la energía… para que el cuerpo se sienta renovado”. Esta percepción se hizo evidente en la forma en que evaluaban la calidad de su sueño, que se basaba más en cómo se sentía su cuerpo al despertar que únicamente en la duración del sueño.

Las prácticas espirituales antes de dormir también desempeñaron un papel importante en las experiencias de sueño de los participantes. Las oraciones y el dhikr —una práctica espiritual islámica que consiste en recordar a Dios mediante oraciones o recitaciones repetitivas— se consideraban útiles para calmar la mente, reducir la ansiedad y facilitar la conciliación del sueño. Un participante explicó: “Rezo para poder dormir profundamente… para que, cuando me despierte, mi cuerpo se sienta bien”. Dichas actividades espirituales formaban parte de sus rutinas nocturnas y se percibían como algo que tenía un efecto calmante. Las percepciones sobre la duración ideal del sueño variaban entre los participantes. Algunas personas mayores consideraban que dormir poco seguía siendo suficiente y no interfería con sus actividades diarias, mientras que otras expresaron el deseo de dormir más tiempo para sentirse más descansadas. Esta variación indicaba que las experiencias de sueño en las personas mayores no siempre se alineaban con los estándares generales del sueño, sino que, por el contrario, estaban influenciadas por los hábitos personales, las condiciones de salud y la tolerancia individual a las necesidades de descanso. La forma en que las personas mayores percibían las funciones del sueño, la duración ideal del sueño y su relación con la salud, tal como se presenta en la Tabla 3, ilustraba cómo los participantes evaluaban el papel del sueño en su vida cotidiana.

### Estrategias Utilizadas Para Manejar los Problemas de Sueño en Personas Mayores

#### Estrategias Conductuales y Rutinas a la Hora de Acostarse

La mayoría de los participantes informaron tener hábitos específicos que les ayudaban a preparar su cuerpo para dormir. Las actividades espirituales fueron las prácticas más mencionadas, entre ellas orar, recitar el dhikr y leer “Yasin” y “Al-Mulk”, que son capítulos del Sagrado Corán que los musulmanes suelen recitar para obtener consuelo espiritual y relajación. Estas actividades se percibían como formas de calmar la mente antes de dormir. Un participante explicó: “Cuando me cuesta dormir, leo y recito el dhikr… al final me quedo dormido por mi cuenta”. Además de las prácticas espirituales, algunas personas mayores realizaban rutinas sencillas como sentarse y relajarse, ordenar la habitación o beber un vaso de leche. Otros mencionaron que veían televisión o usaban sus teléfonos celulares antes de acostarse, especialmente aquellos que vivían con familiares. En algunos casos, sin embargo, el uso de dispositivos retrasaba la hora de acostarse, como lo expresó un participante que dijo: “Hoy en día hay muchos grupos de WhatsApp… así que antes de dormir uso primero mi teléfono”. Estas diversas actividades ilustraron la variedad de formas en que las personas mayores se preparaban para dormir, con opciones que dependían de los hábitos personales, la comodidad y las condiciones de vida cotidianas.

### Estrategias Cognitivas y Emocionales de Afrontamiento

Las personas mayores describieron con frecuencia las estrategias cognitivas y emocionales de afrontamiento como formas de manejar las dificultades para dormir. Los participantes informaron que intentaban calmarse, controlar los pensamientos excesivos y reducir la rumiación antes de dormir. Algunos se recordaban conscientemente “no pensar demasiado”, mientras que otros trataban de redirigir su atención cuando surgían preocupaciones por la noche. La aceptación también se reveló como una importante estrategia de afrontamiento, mediante la cual los problemas de sueño se consideraban una consecuencia natural del envejecimiento. Esta postura ayudaba a reducir la frustración relacionada con los despertares frecuentes o la corta duración del sueño. La afrontamiento emocional estaba estrechamente entrelazado con el significado espiritual. La oración y el dhikr se percibían no solo como comportamientos habituales, sino también como formas de regular las emociones, entregar las preocupaciones y alcanzar la paz mental. A través de este proceso de búsqueda de significado, el sueño se entendía tanto como una necesidad biológica como una fuente de energía renovada y alivio emocional. Estas estrategias cognitivas y emocionales ilustran cómo las personas mayores se adaptaban activamente a la mala calidad del sueño al tiempo que mantenían su bienestar psicológico.

### Estrategias Fisiológicas y de Recuperación

Los adultos mayores también mencionaron estrategias fisiológicas y de recuperación como formas de compensar la mala calidad del sueño nocturno. Varios participantes describieron que recurrían a siestas cortas durante el día para recuperar energía, especialmente cuando no podían volver a dormir después de despertarse por la noche. Las siestas de entre 30 y 60 minutos se percibían como suficientes para reducir la fatiga, aliviar las molestias físicas y mejorar el estado de alerta durante el día. Otros mencionaron que ajustaban sus actividades diarias, como reducir el trabajo físicamente exigente o tomar breves períodos de descanso, para prevenir el cansancio excesivo. Algunos participantes percibían que sus cuerpos se habían adaptado gradualmente a una menor duración del sueño nocturno, lo que sugiere una adaptación fisiológica a los patrones de sueño alterados. Informaron que se despertaban temprano pero que aún se sentían “bien” o sin mareos, lo que indica una variabilidad individual en la necesidad y la tolerancia al sueño. Estas estrategias de recuperación, que incluyen las siestas diurnas, la distribución de las actividades y la adaptación corporal, reflejan los intentos por mantener la capacidad funcional y el bienestar físico a pesar de un sueño nocturno fragmentado o insuficiente.

## DISCUSIÓN

En el presente estudio cualitativo, los principales problemas de sueño reportados por los adultos mayores se relacionaron con la dificultad para conciliar y mantener el sueño, los despertares nocturnos frecuentes y la reducción de la duración total del sueño. Estos problemas estaban estrechamente vinculados no solo a los cambios fisiológicos relacionados con la edad, sino también al malestar psicológico, las preocupaciones familiares y la incomodidad ambiental. Los participantes rara vez mencionaron haber recibido orientación estructurada sobre el sueño por parte de profesionales de la atención de salud o trabajadores comunitarios de la atención de salud, y en general faltaban estrategias formales para el manejo del sueño. Como resultado, las personas mayores recurrían a enfoques por iniciativa propia para lidiar con los problemas de sueño, incluyendo prácticas espirituales, control cognitivo de las preocupaciones, ajustes conductuales y siestas diurnas, con diversos grados de eficacia percibida.

Los hallazgos de este estudio pueden interpretarse desde una perspectiva biopsicosocial del sueño en la tercera edad. En apoyo de esta visión, trabajos anteriores han demostrado que el sueño está integrado en un conjunto más amplio de características biológicas, psicológicas y sociales, y que las personas pueden presentar perfiles biopsicosociales del sueño distintos, en lugar de un único patrón uniforme^(18)^. En nuestro estudio, la calidad del sueño tampoco se percibió únicamente como un proceso biológico o fisiológico, sino como algo moldeado por las experiencias emocionales, las relaciones sociales, las actividades cotidianas y el significado espiritual. La integración de las experiencias de sueño de los participantes, sus estrategias de afrontamiento y las consecuencias durante el día proporcionó una comprensión integral de cómo las personas mayores evalúan su sueño, responden a las dificultades para dormir y valoran el apoyo disponible. Los hallazgos indican la importancia de las estrategias de manejo del sueño que aborden el bienestar psicológico y el contexto social, además de los aspectos fisiológicos del envejecimiento. En consonancia con investigaciones anteriores, las personas mayores reportaron con frecuencia dificultades para conciliar el sueño y mantenerlo, despertares nocturnos frecuentes y una duración del sueño reducida; también se describió con frecuencia el sueño fragmentado, el cual parecía contribuir a un deterioro del funcionamiento diurno^(19)^. En conjunto, estos hallazgos sugieren que los problemas de sueño identificados en este estudio reflejan tanto influencias contextuales locales como cambios más amplios relacionados con la edad documentados a nivel internacional.

Las experiencias relacionadas con el sueño descritas en este estudio estaban profundamente arraigadas en el contexto sociocultural de Indonesia, particularmente en las islas de Bangka Belitung. En este entorno, los fuertes lazos familiares intergeneracionales, las expectativas de piedad filial y la vida comunitaria muy unida determinaban la forma en que se percibían y manejaban los problemas de sueño. Estos hallazgos están respaldados por investigaciones nacionales que muestran que el insomnio entre las personas mayores está asociado con una interacción familiar limitada y con el hecho de vivir solas tras la separación familiar debido a la migración de los hijos^(20)^. Investigaciones previas sobre el cuidado familiar en Indonesia han demostrado que las obligaciones filiales influyen fuertemente en el apoyo a las personas mayores; sin embargo, los cambios socioeconómicos y la migración de los hijos adultos pueden alterar estas redes tradicionales, lo que aumenta la vulnerabilidad y podría contribuir a la alteración del sueño a través del aislamiento o los cambios en los roles familiares^(21)^. Los datos de encuestas nacionales realizadas a personas mayores indonesias también han demostrado que los síntomas de insomnio están asociados con factores sociales y sociodemográficos, entre ellos un bajo nivel educativo, una situación económica precaria, la soledad y una menor satisfacción con la vida, lo que subraya el importante papel que desempeñan los contextos sociales y relacionales en el sueño^(22)^. En conjunto, estos hallazgos sugieren que el sueño en la tercera edad en Indonesia está estrechamente vinculado a las dinámicas familiares y las obligaciones sociales, en lugar de entenderse únicamente como un problema biomédico individual^(23)^. Estos hallazgos sugieren que el sueño en la tercera edad en Indonesia está estrechamente entrelazado con las dinámicas familiares y las obligaciones sociales, en lugar de entenderse únicamente como un problema de salud individual.

Las condiciones ambientales también contribuyeron a la alteración del sueño. Las preferencias en cuanto a la iluminación, la temperatura y el ruido variaron ampliamente, lo que indica que la calidad del sueño está estrechamente vinculada a las percepciones subjetivas de las personas mayores sobre la comodidad de su recámara. En Bangka Belitung, muchos participantes vivían en entornos costeros tropicales caracterizados por altas temperaturas, alta humedad y ventilación limitada. Con frecuencia describían dificultad para conciliar el sueño y despertares nocturnos frecuentes durante las noches calurosas. Estos hallazgos concuerdan con una investigación de campo realizada entre adultos mayores en Shanghái, la cual demostró que las condiciones térmicas y la ventilación del dormitorio están estrechamente asociadas con la calidad del sueño medida objetivamente^(24)^. En ese estudio, tanto una mayor temperatura del aire en el dormitorio como una mayor concentración de CO_2_ se correlacionaron negativamente con los resultados del sueño. Por cada aumento de 1 °C en la temperatura del aire, la eficiencia del sueño disminuyó en aproximadamente un 0,7%, el sueño con movimientos oculares rápidos (REM) se acortó en aproximadamente 2,1 minutos y el tiempo de vigilia aumentó en unos 2,3 minutos. El tiempo total de sueño también disminuyó a medida que aumentaba el CO_2_, con una reducción estimada de alrededor de 11 minutos por cada aumento de 100 ppm^(24)^. Además de las condiciones térmicas, los participantes mencionaron con frecuencia las molestias causadas por la luz y el ruido procedentes de casas cercanas, calles o actividades comunitarias; estudios anteriores han informado de manera similar que la exposición a la luz ambiental y el ruido nocturno están relacionados con la fragmentación del sueño y el retraso en el inicio del sueño en la población de la tercera edad^(25)^.

Un estudio comunitario realizado en Bengala informó que el 87,5% de los adultos mayores experimentaban trastornos del sueño relacionados con los mosquitos durante la noche, y que la mala calidad del sueño era común, ya que el 75% presentaba puntuaciones del PSQI ≥5 y un PSQI global promedio de 8,33, lo que indica que la exposición a los insectos puede ser una fuente ambiental importante de interrupción del sueño en entornos tropicales^(26)^.

La evidencia de grandes cohortes también respalda la contribución de la luz y el ruido a las alteraciones del sueño. En la Cohorte de Adultos Mayores de Yiwu, que incluyó a 2.978 adultos de 65 años o más, el 39,7% fue clasificado como con mala calidad del sueño (PSQI > 5), y tanto la luz nocturna como el ruido se asociaron significativamente con el sueño deficiente, con razones de prevalencia de 1,14 y 1,15, respectivamente. La luz de los televisores o lámparas que se dejan encendidas en la habitación, la luz que ingresa desde el exterior y el ruido del tráfico y de los lugares públicos se asociaron con una mayor latencia del sueño, menor eficiencia del sueño y perturbaciones del sueño más frecuentes^(27)^. En conjunto, estos hallazgos indican que los mosquitos, la luz y el ruido representan factores ambientales significativos que contribuyen a los problemas de sueño descritos por los participantes en este estudio. En conjunto, la convergencia entre los relatos de los participantes y la evidencia previa indica que los factores ambientales como el calor, la humedad, la luz, el ruido y los insectos no son influencias secundarias, sino que están estrechamente entrelazados con la forma en que las personas mayores experimentan y evalúan la calidad de su sueño en la vida cotidiana. Dado que estas condiciones ambientales difieren sustancialmente de las que suelen encontrarse en los países de altos ingresos, también sugieren que las recomendaciones sobre higiene del sueño no pueden simplemente trasladarse desde los contextos occidentales, sino que deben adaptarse a las condiciones de vivienda y a los entornos de vida cotidiana de Indonesia.

Las consecuencias de la falta de sueño se hicieron evidentes en el funcionamiento diurno, ya que los participantes informaron fatiga, somnolencia, menor concentración y disminución de la productividad, lo que confirma que los problemas de sueño afectan no solo al descanso nocturno, sino también a las actividades diarias y a la percepción de la salud. Este patrón concuerda con estudios recientes que muestran que la falta de sueño en las personas mayores se asocia con deterioro cognitivo, declive funcional y menor calidad de vida^(28,29)^. Algunos participantes describieron la percepción de haberse adaptado con el tiempo a una duración del sueño más corta. Este patrón puede interpretarse como una forma de cambio de respuesta, en la que las expectativas y los estándares internos respecto al “sueño adecuado” se recalibran con el envejecimiento, en lugar de reflejar una verdadera mejora en la cantidad o la continuidad del sueño^(30)^. Dicha reinterpretación concuerda con las teorías de la evaluación cognitiva en el envejecimiento, las cuales sugieren que los cambios cognitivos y motivacionales relacionados con la edad influyen en la forma en que las personas mayores evalúan y le dan sentido a sus experiencias —incluidos los síntomas relacionados con la salud— en comparación con sus contrapartes más jóvenes. Por lo tanto, las personas mayores pueden normalizar ciertos cambios, replantearlos como parte del envejecimiento o adaptar estrategias de afrontamiento basadas en estas evaluaciones, tal como lo muestran estudios previos que examinan cómo las personas mayores interpretan y manejan los desafíos estresantes de la vida a través de procesos de evaluación y afrontamiento^(31)^. En respuesta a estas dificultades, las personas mayores adoptaron múltiples estrategias de afrontamiento, entre ellas rutinas previas al sueño, el replanteamiento cognitivo de las preocupaciones, la aceptación emocional y el uso de prácticas espirituales. Estudios cualitativos similares han señalado que las personas mayores suelen recurrir a estrategias conductuales y cognitivas desarrolladas por sí mismas para manejar los síntomas del insomnio^(32)^.

En el presente estudio, estas estrategias se manifestaron no solo a través de ajustes prácticos, como cambiar los horarios diarios o descansar durante el día, sino también mediante formas más amplias de afrontamiento religioso y espiritual. Una revisión de alcance ha indicado que las intervenciones espirituales y religiosas se asocian con mejoras en los trastornos del sueño y los resultados relacionados con el insomnio, lo que sugiere posibles beneficios de las prácticas espirituales para la calidad del sueño^(33)^. De manera similar, un ensayo clínico aleatorizado encontró que los programas de atención espiritual que incorporaban apoyo religioso se asociaban con una mejor calidad subjetiva del sueño. Los estudios centrados en la recitación del Corán han demostrado reducciones en la ansiedad y aumentos en la sensación de calma, lo que puede influir en la forma en que las personas evalúan su sueño^(34)^. Las investigaciones que examinan el dhikr como práctica psicoespiritual entre las personas mayores también han hallado que fomenta la paz interior, reduce la ansiedad y mejora la regulación emocional, aspectos todos ellos relevantes para la experiencia del sueño^(35)^. En general, estas líneas de evidencia sugieren que las estrategias de afrontamiento religiosas y espirituales descritas por los participantes concuerdan con una literatura más amplia que muestra que la espiritualidad puede favorecer el bienestar psicológico y percepciones más positivas del sueño.

Este estudio ofrece información importante sobre cómo las personas mayores perciben la calidad del sueño y hacen frente a los trastornos del sueño en su vida cotidiana. Estos hallazgos concuerdan con estudios cualitativos previos que sugieren que la calidad del sueño no solo depende de factores fisiológicos, sino también de las relaciones sociales, el bienestar emocional, las condiciones ambientales y las creencias culturales. Los hallazgos resaltan que los trastornos del sueño no solo se ven influenciados por condiciones físicas, sino también por factores psicológicos, relaciones familiares, comodidad ambiental y prácticas espirituales^(22)^. Comprender estas experiencias puede ayudar a las enfermeras, los cuidadores y los trabajadores de salud comunitarios a desarrollar intervenciones más individualizadas y culturalmente sensibles para mejorar la calidad del sueño entre las personas mayores. Prestar mayor atención al apoyo familiar, al entorno para dormir y a las estrategias de afrontamiento no farmacológicas, como la relajación y las prácticas espirituales, puede mejorar el manejo del sueño y el bienestar general en la tercera edad. Estos hallazgos también respaldan el papel de los servicios comunitarios para personas mayores, como el Posyandu Lansia, en la promoción de un sueño saludable y la calidad de vida entre las personas mayores.

Se deben reconocer varios puntos fuertes y limitaciones de este estudio. El sueño entre las personas mayores se exploró mediante un enfoque cualitativo a fondo, lo que permitió obtener descripciones detalladas de cómo se experimentaba y se manejaba el sueño en la vida cotidiana. La inclusión de participantes tanto de entornos comunitarios como de atención institucional mejoró la diversidad de perspectivas. El rigor metodológico se respaldó mediante la codificación sistemática, la triangulación y el uso de citas textuales. El estudio también destacó los importantes roles de la espiritualidad y el contexto familiar en la configuración de las experiencias de sueño y las estrategias de afrontamiento. Sin embargo, la muestra relativamente pequeña y seleccionada a propósito de una sola provincia, que no representaba a todas las personas mayores de Bangka Belitung, puede limitar la transferibilidad de los hallazgos y no permite la generalización a toda la población de personas mayores de la provincia ni a otros entornos culturales. Dado que los datos se basaron en experiencias autoinformadas, el sesgo de recuerdo y la deseabilidad social pueden haber influido en los resultados. Además, la calidad del sueño no se midió de manera objetiva, por lo que los hallazgos reflejan parámetros del sueño percibidos y no fisiológicos. A pesar de estas limitaciones, se proporcionó información valiosa sobre el sueño entre las personas mayores y se sentaron las bases para futuras investigaciones.

En resumen, los hallazgos de este estudio indican que el sueño deficiente en las personas mayores es un fenómeno complejo que surge de la interacción entre el envejecimiento biológico, el malestar psicológico, los roles sociales, las condiciones ambientales y el significado espiritual. Las personas mayores no experimentan los problemas de sueño como síntomas clínicos aislados, sino como parte de un contexto de vida más amplio, que abarca las relaciones familiares, las condiciones de salud, las rutinas diarias y las prácticas religiosas. Al mismo tiempo, dependen en gran medida de estrategias que ellos mismos ponen en práctica, con una orientación formal limitada por parte de los servicios de salud, lo que puede hacer que algunos problemas de sueño no se aborden de manera adecuada. Estos resultados subrayan la importancia de adoptar enfoques holísticos y culturalmente sensibles para la atención del sueño de las personas mayores en Indonesia, integrando el apoyo psicológico, la participación familiar y comunitaria, la modificación del entorno y el reconocimiento de la afrontamiento espiritual como recursos legítimos. Estudios futuros deberían examinar cómo dichos enfoques multidimensionales pueden traducirse en intervenciones viables y cómo pueden mejorar la calidad del sueño y el funcionamiento diurno en la tercera edad.

## CONCLUSIÓN

Los hallazgos de este estudio sugieren que las personas mayores de Bangka Belitung experimentaban comúnmente dificultades para conciliar y mantener el sueño, despertares nocturnos frecuentes y una duración corta del sueño, al tiempo que recibían una orientación limitada por parte de los profesionales de la salud. Muchos participantes recurrían a estrategias por iniciativa propia, como prácticas espirituales, control de la ansiedad, cambios de comportamiento y siestas diurnas, las cuales se percibían como de distintos niveles de efectividad y, en algunos casos, como insuficientes para abordar sus problemas de sueño. Estas experiencias resaltan la importancia de un mayor apoyo profesional y de una educación sobre el sueño centrada en el paciente para ayudar a las personas mayores a manejar la mala calidad del sueño y mejorar su bienestar.

## Data Availability

El conjunto completo de datos que respalda los resultados de este estudio se publicó en el propio artículo.
